# Efficient Photocatalytic Degradation of Methylene Blue From Aqueous Solution Using Hybrid Biomass‐Derived Nanostructured Carbon‐TiO_2_ Photocatalyst

**DOI:** 10.1002/chem.202501564

**Published:** 2025-07-17

**Authors:** Aman Sharma, Jyothi Mannekote Shivanna, Navneet Kumar Gupta, Prashanth W. Menezes, Gurumurthy Hegde

**Affiliations:** ^1^ Department of Chemistry Christ University Bengaluru 560029 India; ^2^ Centre for Advanced Research and Development (CARD) Christ University Bengaluru 560029 India; ^3^ Nitte (Deemed to be University), Nitte Meenakshi Institute of Technology (NMIT), Department of Chemistry Bengaluru 560064 India; ^4^ Centre for Sustainable Technologies Indian Institute of Science Gulmohar Marg, Mathikere Bengaluru 560012 India; ^5^ Department of Material Chemistry for Catalysis Helmholtz‐ Zentrum für Materialien und Energie Albert‐Einstein‐Str. 15 12489 Berlin Germany; ^6^ Department of Chemistry Technical Univerisity of Berlin Straße des 17 Juni 135. Sekr. C2 10623 Berlin Germany

**Keywords:** biomass, biowaste‐derived materials, coffee leaves, dye degradation, methylene blue remediation, photocatalysis

## Abstract

Industrial dye usage results in substantial wastewater discharge, posing environmental and health hazards. Hence, developing efficient, sustainable, and cost‐effective treatment technologies is crucial. Photocatalysis using TiO₂ has emerged as a promising approach for dye degradation. This study explores the photocatalytic removal of methylene blue (MB), a model dye pollutant, using a composite of biomass‐derived carbon nanoparticles (CNPs) and nanosized TiO₂ under UV light. The CNPs were synthesized via one‐step pyrolysis from waste coffee leaves, offering a sustainable carbon source. The resulting CNPs (CL‐10) and the TiO₂‐CNP composite (PC@CL‐10) were thoroughly characterized using advanced techniques. Incorporating carbon significantly reduces the band gap of TiO₂ from ∼3.2 eV to 2.90 eV, enhancing photocatalytic activity. Degradation studies under varying catalyst doses, dye concentrations, and pH levels demonstrate effective MB removal under UV irradiation. Photocatalytic experiments revealed up to 99% degradation of MB under UV light, while tests conducted in the dark showed negligible activity, confirming the light‐dependent efficiency. Kinetic analysis indicated that intra‐particle diffusion (IPD) governs the dye degradation process. Moreover, recyclability tests over seven cycles showed consistent performance with minimal decline, highlighting the catalyst's stability and reusability. These findings suggest that PC@CL‐10 is a highly effective, low‐cost photocatalyst with strong potential for large‐scale wastewater treatment applications.

## Graphical abstract

A hybrid photocatalyst of biomass‐derived carbon nanospheres and TiO₂ has been developed from waste coffee leaves, achieving 99% MB dye degradation. The carbon reduces the bandgap, boosting UV photocatalysis, and the material shows excellent stability and recyclability, offering a sustainable, scalable solution for wastewater treatment.

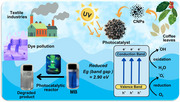

## 1 Introduction

The textile industry is a substantial contributor to water pollution, responsible for around 20% of global water contamination, and ranking as the second‐largest polluting sector after the oil industry.^[^ ^1^ ^]^ Among various pollutants, dyes are particularly challenging to remove due to their chemical stability and complex molecular structures, which make them resistant to biological and photochemical degradation.^[^ ^2^ ^]^ Moreover, many dyes and their degradation products are toxic to aquatic organisms, leading to biodiversity loss and disrupting the ecosystem. Hence, effective treatment of dye‐contaminated water is required to protect marine ecosystems and provide safe water for human consumption.^[^ ^3^ ^]^

Azo dyes are abundant in nature and cause severe contamination in groundwater and rivers that are in the vicinity of dyeing industries. Among them, methylene blue (MB) is one of the most widely used cationic dyes. While it appears green in its solid state at room temperature, it dissolves in water to form a blue solution. However, MB poses a major environmental concern due to its potential toxicity and long‐term persistence in the ecosystems.^[^ ^4^ ^]^ If improperly disposed of, MB waste can severely impact aquatic life and disrupt ecological balance.^[^ ^5^ ^]^ Thus, although MB is involved in industrial processes, environmental impact, and disposal measures are crucial to waste management strategies.

Various techniques are used to remove MB from wastewater, each showing a different degree of success. Most methods usually generate enormous amounts of sludge that necessitate additional treatment and disposal.^[^ ^6^ ^]^ Adsorption is, for example, the most common and very effective way to remove dyes, but it has its usual obstacles of frequently replacing or regenerating costly adsorbents. Furthermore, physical removal methods such as ultrafiltration and reverse osmosis may separate a solution from MB. Nonetheless, aside from being expensive, these techniques are prone to fouling, which leads to decreased performance.^[^ ^7^ ^]^ Biological degradation employs microorganisms to break down MB, but this process is often slow and incomplete, sometimes forming toxic degradation products.^[^ ^8^, ^9^ ^]^ Yet, these methods can be accepted as satisfactory. Still, they possess some significant drawbacks, such as high energy consumption, heavy usage of chemicals, more operational expenses, risks of secondary pollution, decreased efficiency at low dye concentrations, and the need for highly trained staff. Owing to this, there has been an upward trend in attention toward using other approaches, such as photocatalysis, as a more sustainable and efficient alternative to the method of MB degradation.

Photocatalysis is the process through which light‐absorbing catalysts are activated by light and form reactive oxygen species (ROS). These reactive species decompose complex organic molecules into benign byproducts: water and carbon dioxide.^[^ ^10^, ^11^ ^]^ Therefore, the photocatalysis method would be a better alternative than any traditional method.^[^ ^12^ ^]^ It is environmentally sustainable, utilizing light energy to drive the process without generating harmful by‐products, and can fully mineralize organic pollutants.^[^ ^13^, ^14^ ^]^ This method also offers additional benefits, including the reusability of photocatalysts, which helps lower operational costs and its reliance on sunlight, further reducing energy expenses. Moreover, one of the other main advantages of technology is its ability to be functional under ambient conditions, thus promising the potential to be used in the future for mass production.^[^ ^15^ ^]^

Nevertheless, the effectiveness of the whole process largely depends on the photocatalyst‐oriented material. Titanium dioxide (TiO_2_) has been the most thoroughly researched photocatalyst, efficiently breaking down MB in UV exposure.^[^ ^16^ ^]^ The exploration of carbon nanoparticles (CNPs) as photocatalysts for dye degradation using TiO_2_ has garnered significant attention in recent years, mainly due to the pressing need for effective environmental remediation strategies. Carbon‐based photocatalysts, especially those derived from biowaste, have emerged as promising candidates, as they enhance photocatalytic efficiency under visible light.^[^ ^17^ ^]^

Plentiful and sustainable resources such as agricultural residues and food waste can be turned into valuable products with photocatalysis, thus solving the waste disposal problem cost‐efficiently. Primarily, by pyrolysis or carbonization,^[^ ^18^ ^]^ they are generally produced and possess adequate traits for photocatalysis. These materials are usually synthesized via pyrolysis, resulting in ideal properties for photocatalysis. Biowaste‐derived carbon materials offer a sustainable, cost‐effective^[^ ^19^ ^]^ solution for MB degradation and environmental remediation.^[^ ^20^ ^]^ Coffee is the second most traded commodity after oil, generating tons of coffee grounds annually, creating a significant, underutilized resource. Every year, the coffee industry generates more than ten million t of coffee waste globally.^[^ ^21^ ^]^ These residues hold the potential for developing sustainable photocatalysts for dye degradation. The first report on coffee waste to remove pollutants from wastewater was reported in 2002. This work assessed several adsorbents, including coffee bean skins, to remove copper and zinc ions from swine breeding wastewater.^[^ ^22^ ^]^ Among the bioactive compounds found in coffee leaves, caffeine is the most abundant, with a concentration of approximately 24.5 g/kg of dried leaves.^[^ ^23^ ^]^ Coffee waste, being lignocellulosic biomass, is mainly composed of the essential life elements (C, H, O, and N), which primarily form cellulose (59.2–62.94 wt%), hemicellulose (5–10 wt%), and lignin (19.8–26.5 wt%).^[^ ^24^, ^25^ ^]^ In fact, due to their high caffeine content, old coffee leaves may harm the soil and the beneficial microorganisms living in the soil and coffee roots, particularly in large‐scale coffee farming areas. Thus, utilizing coffee leaves for further applications makes better use of available resources while simultaneously mitigating the environmental impacts of coffee leaf waste.

This study uses biowaste coffee leaves to synthesize novel carbon nanoparticles (CL‐CNPs) via pyrolysis at high temperatures. The CL‐CNPs were further used to make a hybrid photocatalyst with TiO_2_. The photocatalytic activity of the prepared photocatalyst PC@CL‐10 was then utilized to degrade cationic MB dye. The effects of operational factors, including the initial MB dye concentration, photocatalyst dose, pH, and irradiation time, were assessed to achieve high performance of the material.

## 2 Results and Discussion

### 2.1 Physicochemical Characterization of Materials

#### 2.1.1 FESEM Analysis

**Figure 1:**
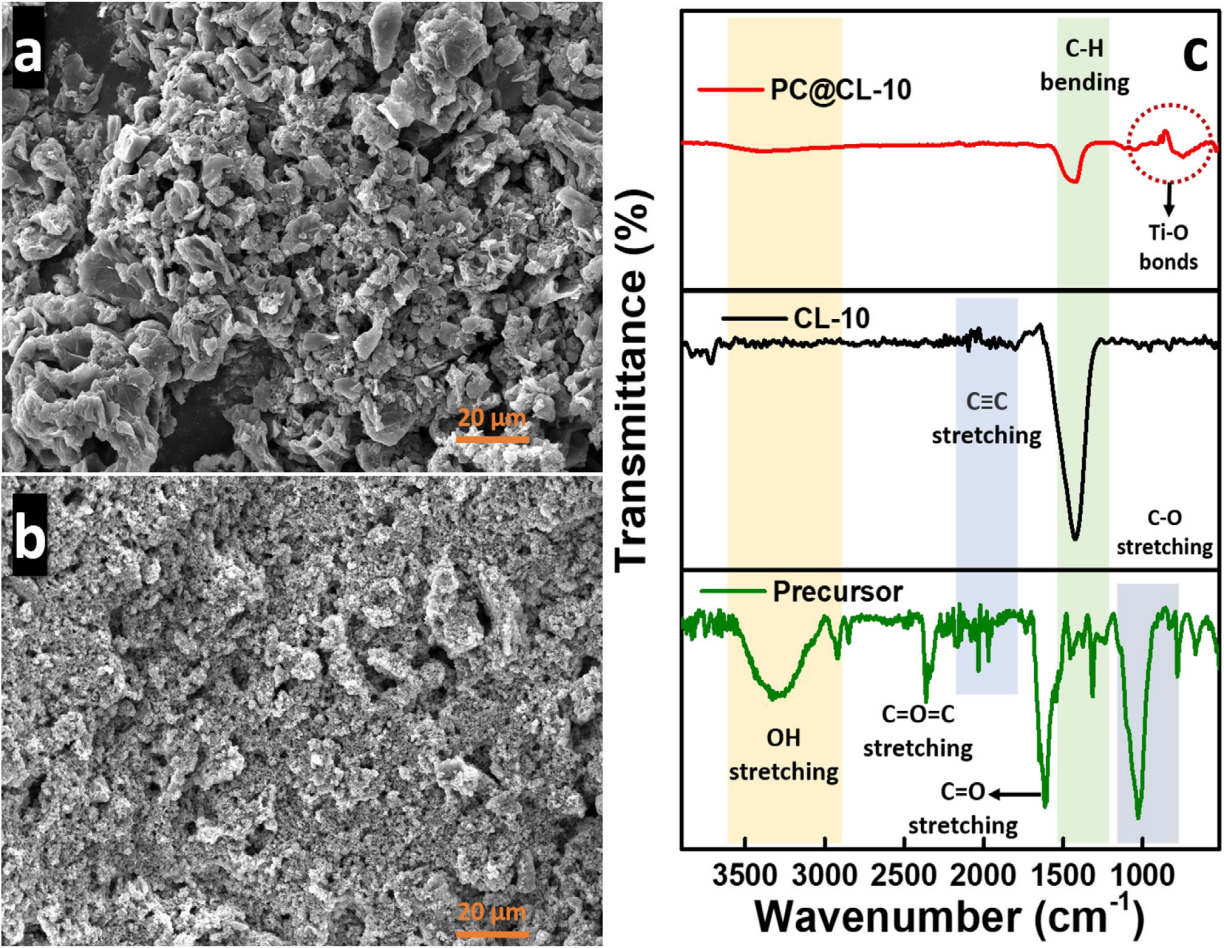
FESEM image of a) CL‐10 and b) PC@CL‐10; c) FTIR spectra of the raw precursor, CL‐10 and PC@CL‐10.

Field emission‐scanning electron microscope (FESEM) analysis of CL‐10 revealed solid, unique structures typical of high‐temperature pyrolyzed carbon materials, as seen in Figure 1a. EDS mapping of CL‐10 shows around 89% of carbon content, and mapping images are shown in the supplementary Figure S1a,b. Rather than existing as separate units, CL‐10 appears to form clusters, contributing to dye removal. The clustering of these particles could be attributed to extended reaction times and gradual cooling from the synthesis temperature to ambient conditions. A more granular and uniform structure can be visualized in Figure 1b for PC@CL‐10. The addition of TiO₂ appears to have modified the texture, resulting in a more compact and densely packed material. The granular surface morphology observed suggests that TiO_2_ particles are primarily distributed on the exterior surface of the CL‐10. The supplementary Figure S1c–e shows the EDS mapping of PC@CL‐10 for the presence of C, O, and Ti.

#### 2.1.2 FTIR Spectra Assessment

Fourier transform infrared (FTIR) spectroscopy confirmed the lignocellulosic nature of the raw precursor (as shown in Figure 1c), where a broad absorption band observed between 3200 and 3500 cm^−1^ is indicative of inter‐ and intra‐molecular O─H stretching vibrations due to the presence of polysaccharides such as cellulose, hemicellulose, and polyphenolic compounds. The 2900–3000 cm^−1^ absorption peaks correspond to C─H stretching, typically associated with aliphatic hydrocarbon chains. A pronounced peak around 1700 cm^−1^ signifies C═O stretching, generally attributed to carbonyl‐containing functional groups such as carboxylic acids, esters, or aldehydes. Additionally, the bands between 1000 and 1200 cm^−1^ indicate C─O stretching vibrations, which may originate from lignocellulosic components in coffee leaf powder.^[^ ^26^ ^]^ As seen in FTIR spectra for CL‐10, the broad O─H band region is notably reduced, indicating the loss of hydroxyl groups during pyrolysis. Multiple smaller peaks around 2100 cm^−1^ suggest C≡C stretching. The sharp peak observed at 1450 cm^−1^ is attributed to aromatic C‐H stretching, signifying the formation of aromatic carbon structures during carbonization. Other minor peaks within the 1000–1300 cm^−1^ range may correspond to residual C─O stretching, though they appear diminished compared to the precursor, confirming the formation of graphitic CNPs. In the FTIR spectra of the photocatalyst, a new band around 667 cm^−1^ confirms the presence of TiO_2_, as it corresponds to Ti─O─Ti stretching vibrations. The broad peak in the corresponding O─H vibrations around the 3400 cm^−1^ range slightly re‐emerges, likely due to hydroxyl groups and absorbed water on the TiO₂ surface.^[^ ^27^ ^]^ The peaks near 1600 cm^−1^ suggest the retention of aromatic C═C bonds, indicating that the graphitic carbon backbone remains intact. Additionally, the 1000–1200 cm^−1^ region may exhibit overlapping signals from C─O stretching and Ti─O bonds.^[^ ^28^ ^]^ The decrease in the intensity of organic peaks, such as C═O and C─H, as seen in CL‐10, suggests structural modifications following TiO₂ incorporation.

#### 2.1.3 BET Analysis

**Figure 2:**
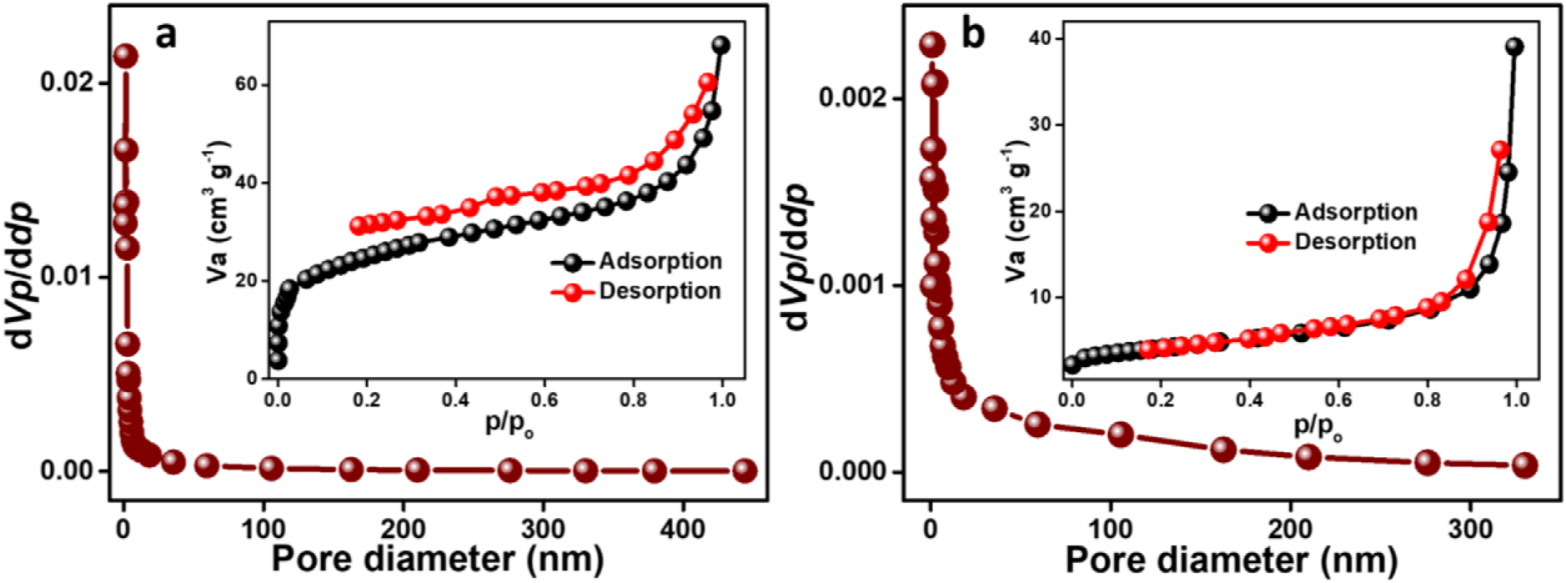
BJH pore distribution plot for a) CL‐10 (inset BET N_2_ adsorption‐desorption curve of CL‐10) and b) PC@CL‐10 (inset BET N_2_ adsorption‐desorption curve of PC@CL‐10).

Brunauer‐Emmett‐Teller (BET) N_2_ adsorption‐desorption isotherms and Barrett‐Joyner‐Halenda (BJH) pore size distribution curves for CL‐10 (Figure 2a) and PC@CL‐10 (Figure 2b) reveal significant structural changes upon TiO₂ incorporation. Both exhibit Type IV isotherm with H2 hysteresis loop, a mesoporous material characteristic. However, it can be observed that PC@CL‐10 shows a less pronounced hysteresis loop, which might be due to the partial blocking of mesopores by TiO_2_. CL‐10 features a network of interconnected mesopores, indicating a well‐defined mesoporous structure with an average pore diameter of 4.5 nm, a BET surface area of 86.4 m^2^/g, and a pore volume of 0.98 cm^3^/g. The hysteresis loop reveals signs of pore blocking and delayed capillary condensation, further validating the intricate pore network within the material. PC@CL‐10 also displays a notably smaller surface area of 14.68 m^2^/g. However, it has a larger pore diameter of 14.2 nm and a significantly reduced pore volume of 0.05 cm^3^/g. The less pronounced hysteresis loop suggests a decrease in mesoporosity, likely due to the TiO₂ nanoparticles partially obstructing the mesopores during synthesis. This structural modification reduces nitrogen adsorption but does not affect the photocatalytic performance of the material in dye degradation. Photocatalysis worked well because the TiO₂‐carbon interaction improved charge transfer, light absorption, and ROS generation, making degradation efficient even with reduced surface area.

#### 2.1.4 XRD Pattern Analysis

**Figure 3:**
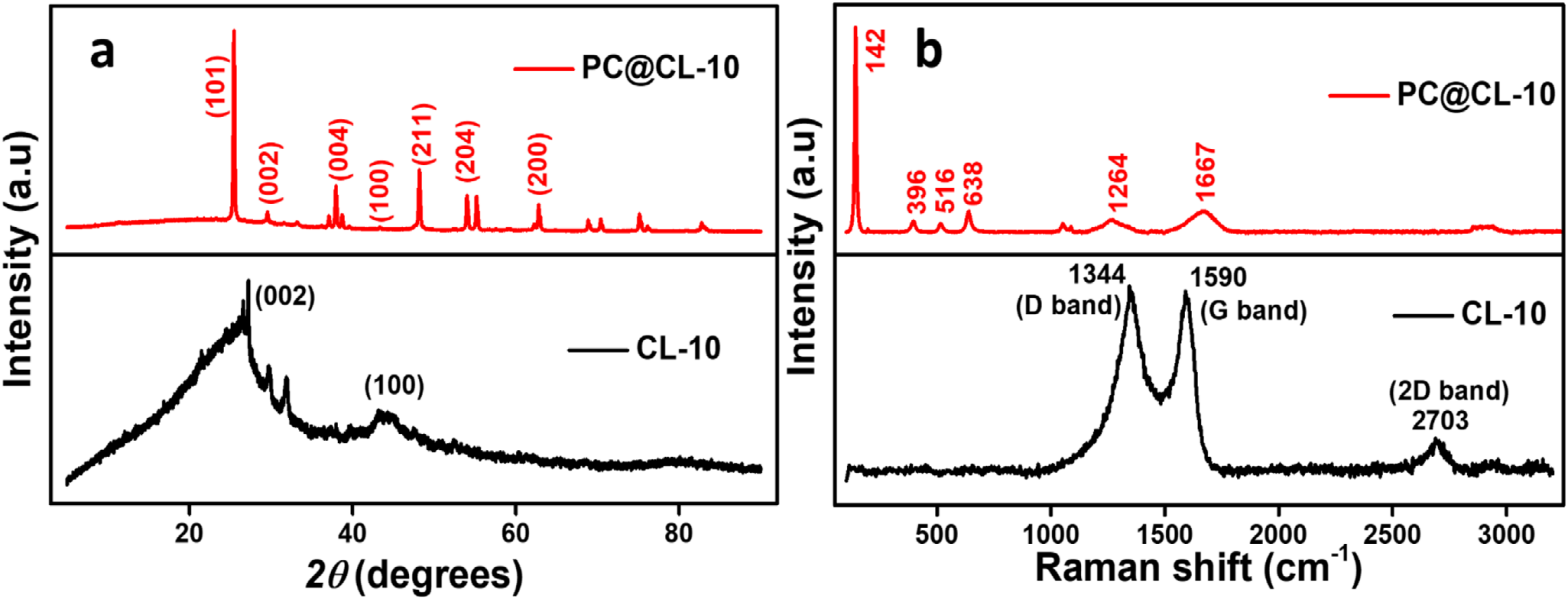
a) XRD spectra of CL‐10 and PC@CL‐10; b) Raman spectra of CL‐10 and PC@CL‐10.

The X‐ray diffraction (XRD) pattern of the synthesized CL‐10 showed two characteristic peaks of carbon nanospheres at 2θ = 26° and 44° corresponding to the (002) and (100) planes, respectively. The broad peak confirms the amorphous nature of CL‐10 and the disorderliness in the graphitic carbon structures.^[^ ^20^, ^29^ ^]^ There are two minor peaks at 29° and 31°, indicating the presence of silica residue or carbon impurities. This is indicative of the carbon synthesized at higher pyrolysis temperatures. The XRD spectra of PC@CL‐10, as shown in Figure 3a, indicate sharp peaks at 2θ = 25.3°, 37.8°, 48.0°, 54.1°, and 62.7° which are ascribed to the anatase phase of TiO_2_ (JCPDS 21–1272). The sharpness in the peak confirms the high crystallinity of the prepared photocatalyst.^[^ ^30^ ^]^ The minor peak around 29° and 44° is attributed to a carbon backbone that also overlaps with a peak at 25.3°, affirming the coexistence of graphitic‐like carbon nanospheres and TiO_2_. The CL‐10 supports TiO₂, potentially enhancing charge transfer and preventing recombination of photogenerated electron–hole pairs during photocatalytic reactions of PC@CL‐10.

#### 2.1.5 Raman Spectra Analysis

Raman spectra, shown in Figure 3b, align with the XRD data that CL‐10 is amorphous in nature. The D band at 1344 cm^−1^ is due to the defects in the carbon lattice, which corresponds to the disordered carbon structure. The G band at 1590 cm^−1^ is due to the Eg mode of graphitic domains in the sp^2^ (C═C) hybridized carbon. There is another characteristic peak of graphitic materials corresponding to a 2D overtone at 2703 cm^−1^.^[^ ^31^ ^]^ The intensity ratio (ID/IG) was found to be 1.03, which indicates a higher level of structural disorder defects and the amorphous character of CL‐10. The characteristic peaks of TiO_2_ additionally appeared in the Raman spectra of the photocatalyst. The carbon backbone (1264 and 1667 cm^−1^) intensity was prominently decreased in the Raman signal of PC@CL‐10, representing surface modifications on TiO_2_. The peaks at 142 (Eg mode), 396 (B1g mode), 516 (A1g mode), and 638 cm^−1^ (Eg mode) correspond to the vibrational modes^[^ ^32^, ^33^ ^]^ of TiO_2_ incorporated in PC@CL‐10.

#### 2.1.6 XPS Analysis

**Figure 4:**
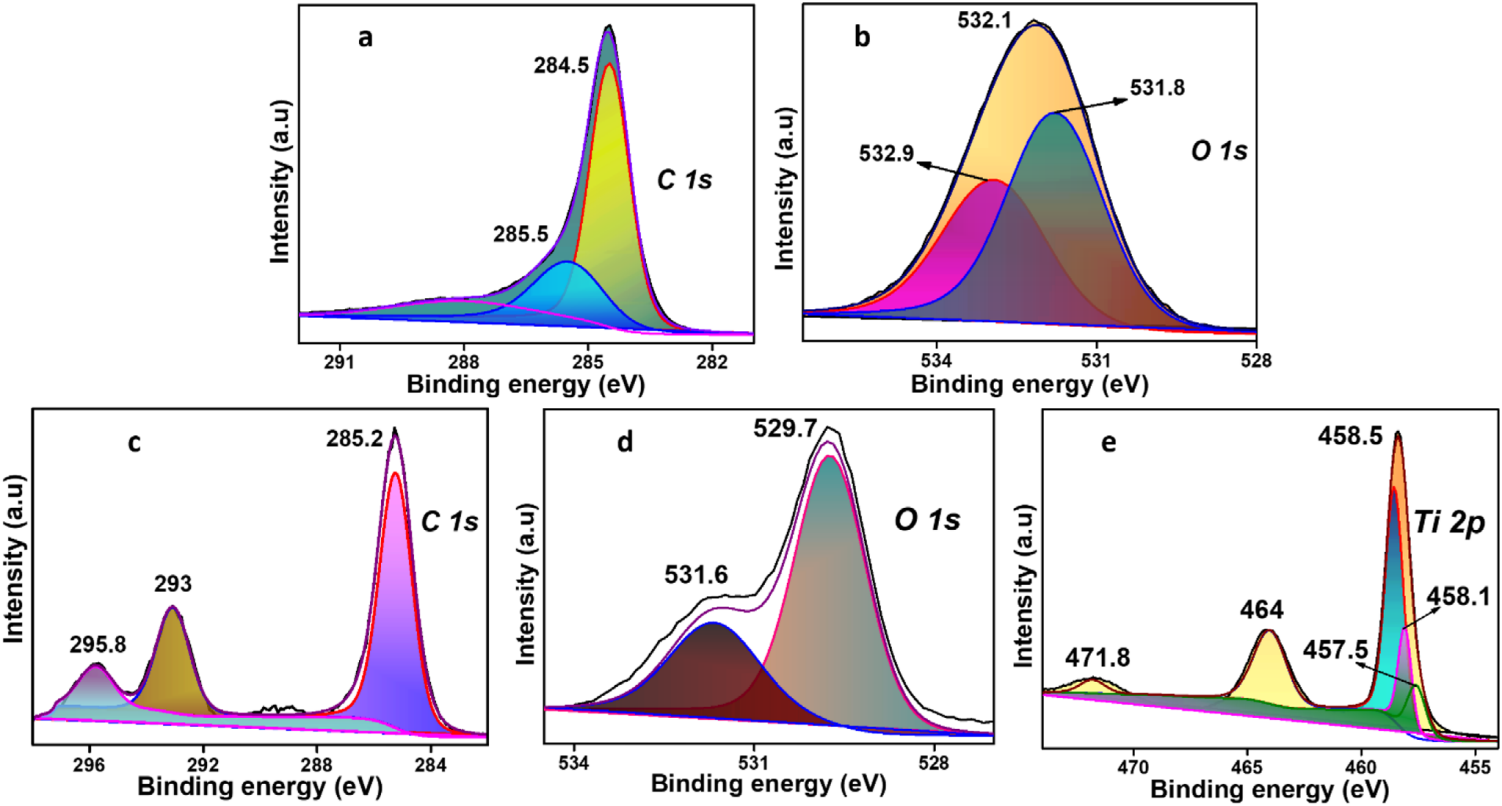
XPS deconvoluted spectra of a,b) CL‐10, and c–e) PC@CL‐10.

The X‐ray photoelectron spectroscopy (XPS) survey spectrum (refer to supplementary Figure S2a) of CL‐10 mainly features a strong C 1 s peak, confirming carbon as the primary element. Smaller O 1 s peaks also appeared as oxidation occurred, indicating surface functional groups that align with the FTIR data obtained. Figure 4a shows C 1 s spectra with a dominant peak with a larger proportion at 284.5 eV corresponding to the C═C bond of sp^2^ hybridized carbon and another peak at 285.5 eV corresponding to C─C bond formation^[^ ^34^ ^]^ in the synthesized material. Figure 4b confirms O═C bonds at 532.9 and 532.1 eV binding energies. The peak at 531.8 eV confirms the presence of O─C bonds in CL‐10.^[^ ^35^ ^]^ XPS survey spectrum of PC@CL‐10 (refer to supplementary Figure S2b) confirms C 1 s, O 1 s, and Ti 2p peaks, and respective deconvoluted binding energy spectra are plotted in Figure 4c–e. The C 1 s spectra (Figure 4c) of PC@CL‐10 show a prominent peak at 285.2 eV corresponding to C─C bond formation, also present in CL‐10. A minor peak at 293 and 295.8 eV corresponds to π–π stacking (C═O), indicating the sp^2^ hybridized carbon structures present in the photocatalyst. Figure 4d shows a binding energy peak at 529.7 eV, characteristic of Ti─O bond formation in the photocatalyst. The minor peak at 531.6 eV might be associated with the hydroxyl group adsorbed to oxygen functionalities of TiO_2_.^[^ ^36^ ^]^ Figure 4e shows the deconvoluted spectra of Ti 2p of the photocatalyst, from which a prominent peak at 485.5 eV suggests Ti 2p_3/2_ and 464 eV confirms Ti 2p_1/2_ state with Ti^4+^ oxidation state of the TiO_2_. Additionally, peaks at 457.5 and 458.1 eV suggest that Ti 2p_3/2_ demonstrates the presence of Ti^3+^ in the photocatalyst, enhancing the photocatalytic degradation of dyes.

#### 2.1.7 Photoluminescence Analysis

The photoluminescence (PL) spectra of the synthesized materials reveal critical insights into the charge carrier dynamics. CL‐10 exhibits lower‐intensity emission peaks at 394 nm and 417 nm. PC@CL‐10 exhibits strong emission peaks around 404 nm and 427 nm, which are typically attributed to surface defects and oxygen‐related vacancies. Although high PL intensity in the PC@CL‐10 composite might typically suggest increased electron–hole recombination, it does not necessarily indicate poor charge separation in this case. The material still exhibits strong photocatalytic activity, demonstrating that the photoexcited electrons actively contribute to dye degradation despite some radiative recombination. This can be attributed to several factors. TiO_2_ inherently possesses excellent photocatalytic properties under UV light, enabling efficient electron–hole generation and participation in surface reactions when added to CL‐10. The presence of carbon nanospheres likely enhances dye molecule adsorption, increasing the proximity of pollutants to reactive sites. It is important to note that although PC@CL‐10 shows slightly higher PL intensity than CL‐10, CL‐10 itself has minimal photocatalytic activity and weak luminescence due to its amorphous carbon structure. Thus, the relevant and appropriate comparison for recombination suppression is between TiO_2_ and CL‐10. Additionally, a bathochromic shift of ∼10 nm was observed in the PL peak of PC@CL‐10 relative to CL‐10, suggesting electronic interaction and band structure modification upon TiO_2_ incorporation into CL‐10. The bandgap for PC@CL‐10 was found to be 2.90 eV from the plot (refer to supplementary Figure S3 for PL spectra). This further indicates the formation of a heterojunction and supports the hypothesized electron transfer mechanism from TiO_2_ to carbon, enhancing the photocatalytic performance. These combined effects suggest that the overall photocatalytic efficiency of PC@CL‐10 is governed not only by charge carrier dynamics but also by synergistic interactions, enhanced surface properties, and broader light absorption.^[^ ^37^ ^]^

### 2.2 Effect of Different Parameters on Photocatalytic Degradation of MB

#### 2.2.1 Degradation Studies in Dark and Light Illumination

**Figure 5:**
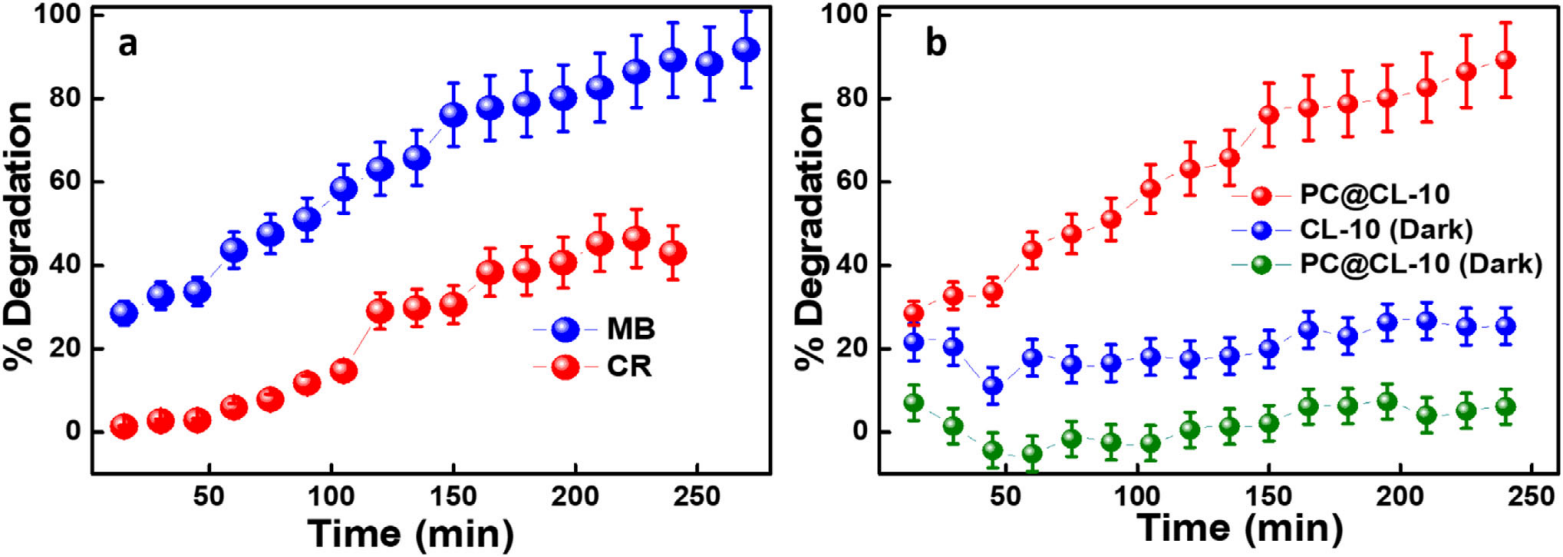
a) Contact time studies for degradation of MB and CR dyes using PC@CL‐10; b) Comparison of PC@CL‐10 and CL‐10 in the dark and with UV irradiation (Dye concentration‐ 5 ppm; Dosage‐ 0.1 g/L; pH‐Native).

The photocatalytic performance of PC@CL‐10 (0.1 g/L dosage) was evaluated under both dark and UV light conditions using MB dye (5 ppm concentration) as a model pollutant. Under dark conditions, the composite showed minimal activity, with only ∼6% degradation after 240 minutes, indicating that light activation is essential for initiating photocatalysis. Figure 5b further confirms that the dye concentration remained nearly constant in the absence of light, emphasizing the photo‐dependent nature of the catalyst. In contrast, upon UV illumination, PC@CL‐10 achieved a significant enhancement in degradation efficiency, reaching 90% removal of MB within 240 minutes, demonstrating its excellent photocatalytic potential. Although pristine TiO₂ reached the same degradation level (refer to supplementary Figure S4b) within a shorter duration (60 minutes), its higher rate can be attributed to the abundance of active photocatalytic sites. However, the slower kinetics observed in PC@CL‐10 are compensated by the added benefits of cost‐effectiveness and sustainability, as the CNPs are synthesized from renewable biowaste and reduce the required TiO_2_ content in the composite. Additionally, standalone carbon nanospheres (CL‐10) exhibited notable adsorption capability, removing approximately 25% of the dye in dark conditions and around 30% dye degradation in light over 240 minutes. This highlights their potential as efficient adsorbents. Integrating CL‐10 with TiO₂ enhances photocatalytic efficiency and introduces dual functionality– adsorption and photodegradation, making PC@CL‐10 a highly promising material for sustainable and large‐scale wastewater treatment. (refer to supplementary Figure S4 for the bar graph indicating degradation efficiency in dark and UV irradiation) Table S1 presents a comparative literature review of different carbon‐TiO_2_‐based photocatalysts for dye degradation. However, this is the first report on utilizing CNPs synthesized via a one‐step pyrolysis method from coffee leaves and utilizing them for photocatalytic degradation by combining them with TiO_2_.

#### 2.2.2 Effect of Contact Time on MB Degradation Using PC@CL‐10

The contact time investigation is an important characteristic that indicates the actual time required for photocatalytic dye degradation at different dye concentrations. The effect of contact time was analyzed using a 5 ppm solution of MB and 0.1 g/L of PC@CL‐10 concentration from 0 to 270 minutes. The degradation experiment was carried out under constant UV light irradiation in a photocatalytic chamber. Figure 5a shows that MB (cationic) dye degradation is much faster than the CR (anionic) dye due to the surface charge properties and other interactions between the photocatalyst and dye. MB degraded by over 90% within 240 minutes, whereas CR dye degraded by 43%. As reported in other studies, a gradual increase in photodegradation was noted with an increase in time. The early phases of MB dye degradation are faster due to more surface active sites on the photocatalyst, as confirmed by the FESEM‐EDS analysis, which shows that TiO_2_ is deposited onto the carbon framework. These surface‐active spots stimulate the catalyst to break down faster.^[^ ^38^ ^]^ However, with the increase in time and higher dosages of photocatalysts, CR degradation would also be possible. For this study, MB dye degradation was studied in detail. (refer to supplementary Figure S5 for the degradation efficiency bar graph at contact time for the effect of photocatalyst dosage, dye concentration, and pH after 240 minutes of UV light irradiation)

#### 2.2.3 Effect of Photocatalyst Dosage on MB Degradation Using PC@CL‐10

Dye degradation is highly influenced by photocatalyst concentration. The photodegradation of dye accelerates as the amount of photocatalyst increases. Several works have shown the influence of catalyst loading on the photodegradation of dyes in wastewater.^[^ ^39^, ^40^, ^41^ ^]^ As the catalyst concentration increases, the number of active sites on the surface of the photocatalyst increases. As a result, the production of OH radicals increases, which is responsible for the discoloration of the dye solution. Beyond a specific catalyst concentration, the solution becomes cloudy, blocking the UV rays required for the reaction and resulting in a lower degradation percentage.^[^ ^42^ ^]^ Adsorption of cationic dyes on oxide surfaces is favored, increasing their concentration and facilitating photocatalytic degradation.^[^ ^43^, ^44^ ^]^

**Figure 6:**
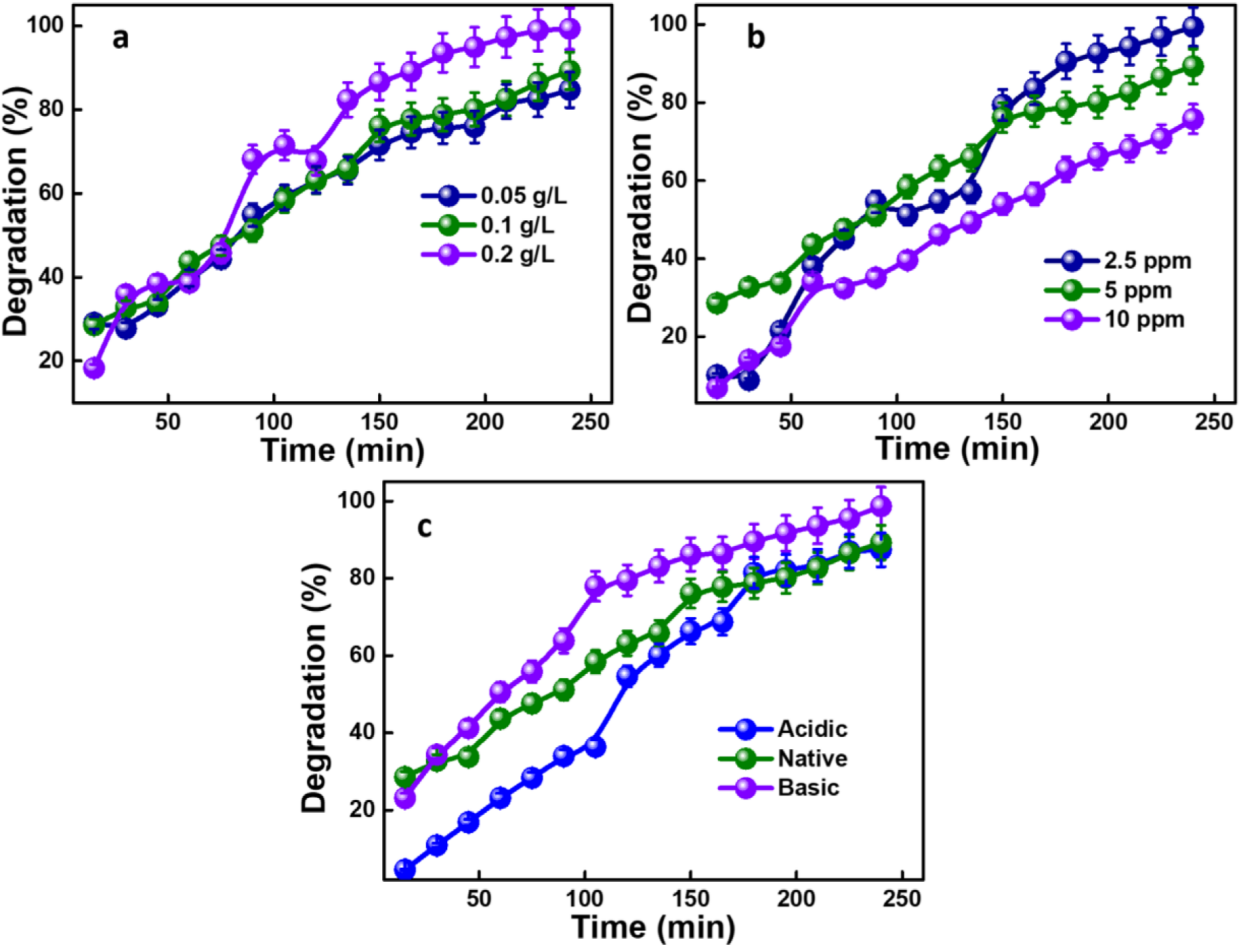
Effect of a) PC@CL‐10 dosage; b) dye concentration; c) pH on MB degradation using PC@CL‐10 over 240 minutes at every 15‐minute interval.

The higher the surface area of the catalyst, the better the adsorption of MB in aqueous suspension.^[^ ^45^ ^]^ Figure 6b shows that the degradation efficiency gradually increases with the increase in photocatalyst (PC@CL‐10) concentration in the MB dye solution. At 240 minutes, 85%, 90%, and 99% degradation efficiencies were observed for PC@CL‐10 concentrations of 0.05 g/L, 0.1 g/L, and 0.2 g/L, respectively. As discussed in previous literature, the increase in degradation efficiency with higher concentrations of PC@CL‐10 can be attributed to the greater availability of active catalytic sites at higher dosages.^[^ ^46^ ^]^ With a concentration of 0.05 g/L of PC@CL‐10, the availability of active sites is limited, which hinders the interaction of the dye molecule and the photocatalyst. When the concentration is further increased to 0.1 g/L and 0.2 g/L, the number of active sites grows by the same measure, thus allowing more dye molecules to interact with the catalyst, thereby increasing the degradation rate. At 0.2 g/L, the catalyst concentration likely provides an optimal balance between available active sites and pollutant molecules, achieving nearly complete degradation. An increasing catalyst loading increases the number of photons absorbed and, thus, the degradation rates.^[^ ^47^ ^]^ However, further increases in concentration beyond this point could lead to diminishing returns due to factors such as light scattering or reduced penetration depth, which may affect the efficiency of the photocatalyst. Although at lower dosages, sufficient degradation efficiency is seen in the MB dye.

#### 2.2.4 Effect of Initial Dye Concentration on MB Degradation Using PC@CL‐10

The effect of initial MB concentrations on the photocatalytic efficiency of PC@CL‐10 was calculated by varying concentrations of MB dye while maintaining a catalyst loading of 0.1 g/L. The degradation efficiency decreases with increasing initial concentrations, as shown in Figure 6a. The decolorization rate was determined by the formation of OH radicals on the catalyst surface and their reaction with dye molecules. Increasing the initial amount of dye led to more accessible molecules for excitation and energy transfer.^[^ ^48^ ^]^ This is likely due to the increased interaction between dye molecules and the catalyst surface, along with a higher number of dye molecules in the solution. Thus, the active sites on the catalyst are covered by dye molecules, which, in essence, interrupt the photons and hence lower the activity of the catalyst. Thus, more oxygen radicals are generated on the surface of the photocatalyst.^[^ ^49^, ^50^ ^]^ At a lower concentration of 2.5 ppm, within 180 minutes, greater than 91% degradation efficiency was observed, and in 240 minutes, 99% degradation was observed. At a higher concentration of 10 ppm, 76% degradation was observed with 240 minutes of light irradiation. The higher dye concentration also shields the UV light.^[^ ^47^ ^]^ This lowers the path length of the photons that enter the solution.^[^ ^51^ ^]^ At 5 ppm, the efficiency was a 90% degradation rate. Since the CL‐10 used to prepare the photocatalyst is prepared by an eco‐friendly process, and the precursor used is from a biomass source, the whole process is sustainable. Therefore, if a higher photocatalyst dosage to a saturation limit is used, the time can be reduced further. A higher dye concentration with a higher photocatalyst dosage can further increase the degradation efficiency.

#### 2.2.5 Effect of pH on MB Degradation Using PC@CL‐10

pH is an essential factor in the degradation of the dyes. The prepared photocatalyst PC@CL‐10 can degrade the MB dye at all pH levels. TiO_2_ has an amphoteric feature, which allows for the development of either a positive or negative charge on its surface.^[^ ^52^ ^]^ MB dye is cationic, and the degradation efficiency is high at a basic pH.^[^ ^53^ ^]^ At 180 minutes, the degradation efficiency was nearly 90%, and at a higher contact time of 240 minutes, it was 99%. Previous research shows that the basic pH electrostatic interactions between negative PC@CL‐10 and MB cations result in significant adsorption and a rapid degradation rate.^[^ ^41^ ^]^ The high proton concentration in acidic solutions slows down the photodegradation of the dye, resulting in reduced efficiency. In an alkaline medium, however, hydroxyl ions neutralize the acidic end products produced by the photodegradation mechanism.^[^ ^54^ ^]^ Photocatalytic degradation in an acidic (pH = 3) environment was also investigated, where it had minimal effect with a degradation efficiency of 87%. At the native pH of the dye solution, the degradation was 90% (Figure 6c). It is clear from the investigated experiments that basic pH is also favored for MB degradation, thereby indicating the stability of the synthesized photocatalyst under various pH conditions.

#### 2.2.6 Reusability Studies

**Figure 7:**
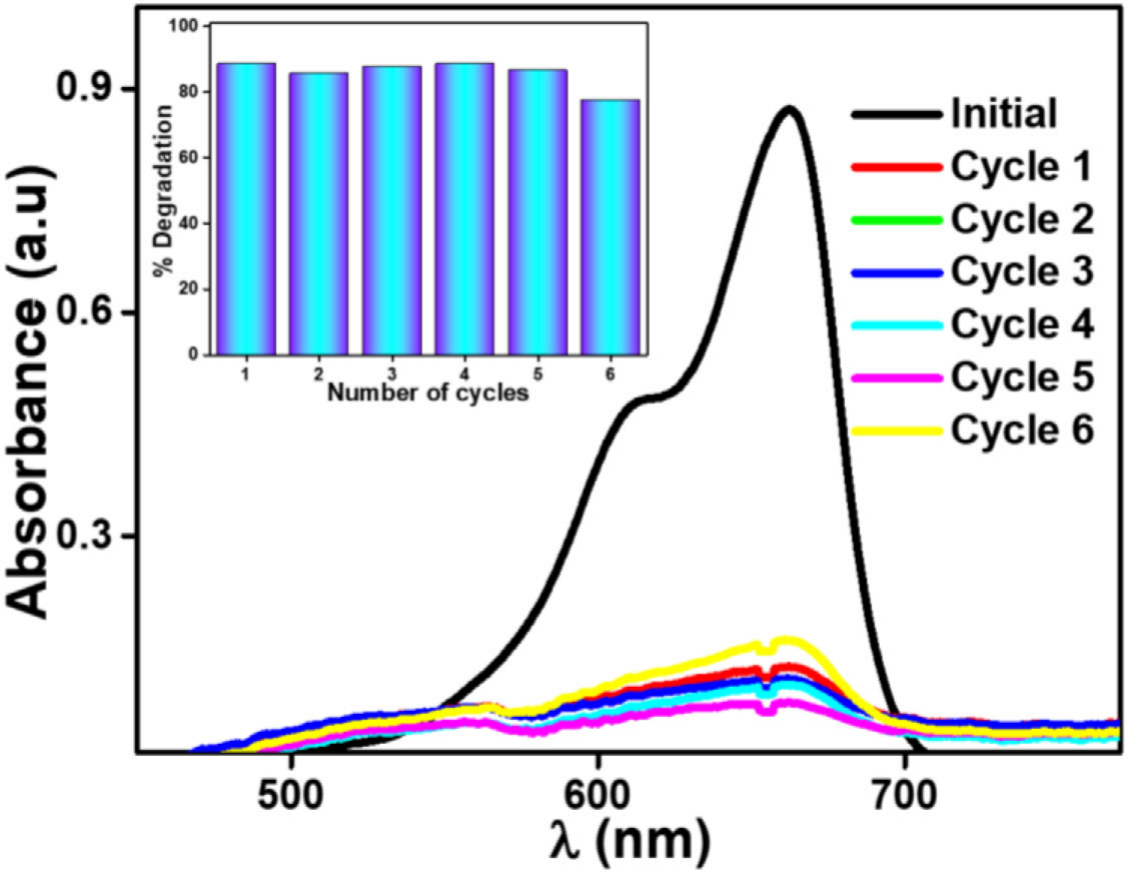
UV‐Vis absorbance plot for recyclability and reusability test of PC@CL‐10 using MB dye for multiple cycles under UV light irradiation for 240 minutes (inset is the bar graph indicating degradation percentages across cycles).

The capacity to reuse a photocatalyst is essential for cost‐effective and environmentally sustainable industrial applications. Studies were conducted to evaluate the potential for reusing the spent photocatalyst in multiple cycles to enable their recycling. The recovered photocatalyst was thoroughly washed with ethanol and distilled water in the initial cycle. After drying, they were reused for a second cycle. This process was repeated for up to six cycles using MB dye. The degradation efficiency remained above 90% in the initial cycles but gradually decreased with repeated reuse.^[^ ^55^ ^]^ By the sixth cycle, the efficiency slightly declined, reaching 78%. The reusability data is presented in Figure 7. After repeated photocatalytic cycles, a decline in TiO₂ activity is often observed, particularly after the sixth cycle. This reduction can be linked to multiple factors. Prolonged UV exposure can trigger partial photocorrosion in TiO₂, leading to the formation of reactive peroxo species that degrade its crystalline integrity and active surface. Simultaneously, the accumulation of dye degradation by‐products may cause surface fouling and pore blockage, especially in mesoporous structures like CL‐10, hindering access to active sites. Additionally, repeated recovery steps such as centrifugation and drying can lead to nanoparticle aggregation, reducing surface area, light absorption, and charge transfer efficiency, ultimately compromising photocatalytic performance, as also reported in previous research.^[^ ^56^, ^57^ ^]^

#### 2.2.7 Dye Degradation Kinetics

**Figure 8:**
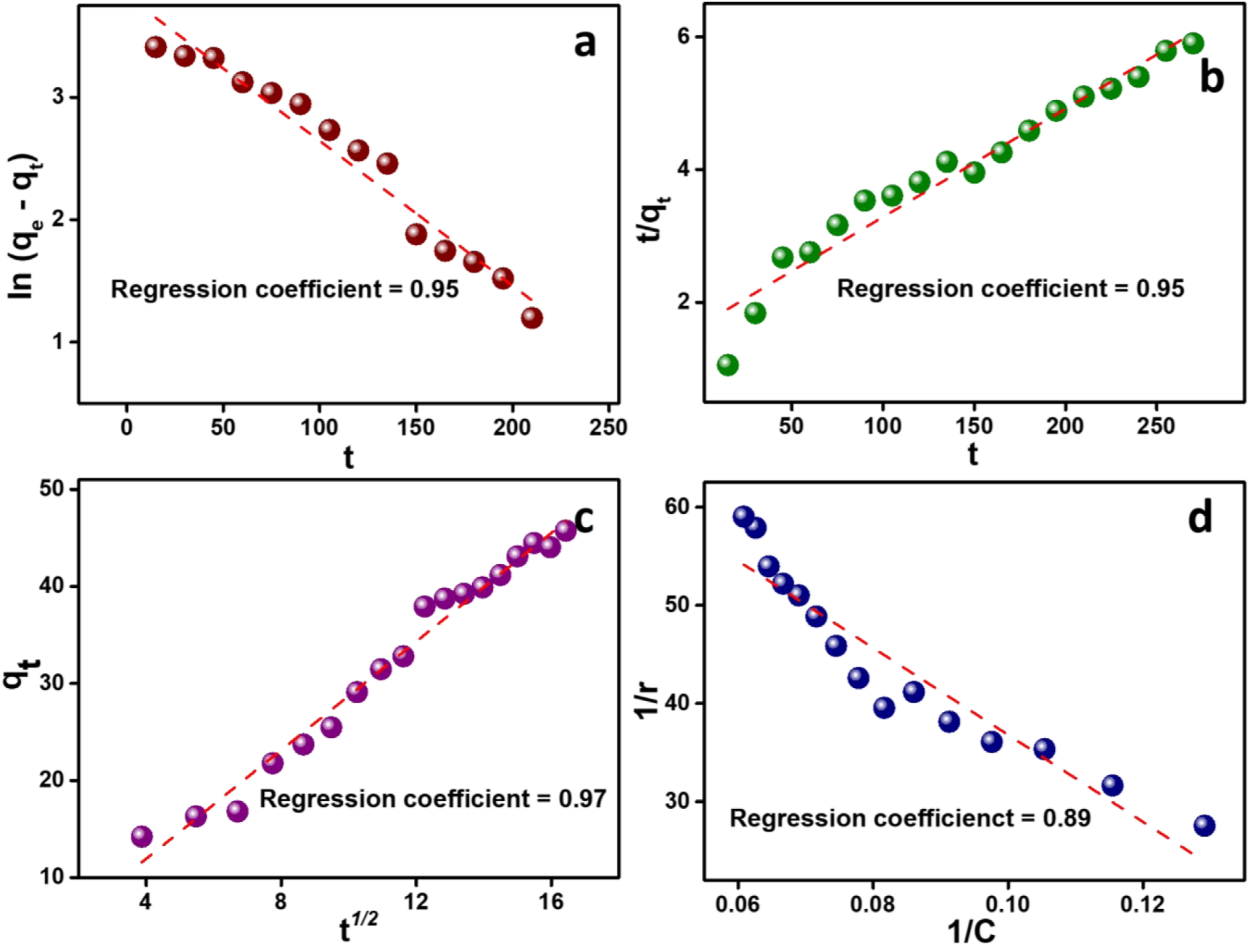
Linear fit plots of a) Pseudo‐First‐Order kinetic model; b) Pseudo‐Second‐Order kinetic model; c) Intra‐particle diffusion model; d) Langmuir‐Hinshelwood model for photocatalytic degradation of MB using PC@CL‐10.

Kinetic models are necessary for analyzing photocatalytic degradation since they help to understand the reaction processes and rate‐determining phases. The fitted plots of these kinetic models are shown in Figure 8a–d. The corresponding model fitting parameters are summarized in Table 1. The four kinetic models used were Pseudo‐first‐order (PFO), Pseudo‐second‐order (PSO), Intraparticle diffusion (IPD), and Langmuir‐Hinshelwood (L–H) kinetic models. The regression (correlation) coefficients (R^2^) were used to assess the validity of each model.

**Table 1:** Kinetic parameters for MB dye degradation using PC@CL‐10 based on different kinetic models.

PFO		PSO		IPD		L–H	
**k_1_** **(minute^−1^)**	0.03	**k_2_ (g mg^−1^minute^−1^)**	2269.49	***K*_diff_ (mg g^−1^.minute^1/2^)**	2.79	**K (L mg^−1^)**	5.46
***q*_e_ (mg g^−1^)**	51.34	***q*_e_ (mg g^−1^)**	61.31	**C (mg g^−1^)**	0.75	**k_r_ (L mg^−1^.minute)**	0.01
**R^2^**	0.95	**R^2^**	0.95	**R^2^**	0.97	**R^2^**	0.89

#### 2.2.8 Pseudo‐first‐order (PFO) Kinetic Model

The PFO kinetics model in dye photodegradation of MB dye illustrates how the degradation rate is related to the dye concentration in the solution at any given time. It is assumed that degradation is predominantly determined by surface reactions, such as dye molecule adsorption onto the photocatalyst. The following linear fit equation 1 is applied to check its fit in the photocatalytic degradation of dye.^[^ ^58^ ^]^

(1) $$\log\left(q_{e}-q_{t}\right)=\log q_{e}-\frac{k_{1}t}{2.303}\;$$

where, *q*_e_ and *q*_t_ represent the amounts of adsorbate adsorbed at equilibrium and at time (t), respectively, while *k*_1_ (minute^−1^) denotes the rate constant.

A linear fit plot of ln (*q*_e_–*q*_t_) versus time is obtained as plotted in Figure 8a. Subsequently, the R^2^ value of 0.95 is obtained by linear fit, indicating a high correlation with this model. A moderate rate constant k_1_ (0.03 minutes^−1^) shows stable photocatalytic activity, although if a higher value were obtained, it would suggest much faster photodegradation. The value of *q*_e_ = 51.34 mg g^−1^ represents the maximum quantity of MB dye adsorbed on the PC@CL‐10 at equilibrium. This shows that the PC@CL‐10 has a high capacity for dye adsorption, which is critical in the overall degradation process.

#### 2.2.9 Pseudo‐second‐order (PSO) Kinetic Model

The PSO kinetics model assumes that chemical interactions (chemisorption), such as electron sharing or transfer between the dye and the active sites of the photocatalyst, determine the degradation rate.^[^ ^59^ ^]^ The following linear fit equation 2 is used to assess the correlation of the PSO model.^[^ ^20^ ^]^

(2) $$\;\frac{1}{q_{t}}=\frac{1}{k_{2}q_{e}^{2}}+\frac{1}{q_{e}}t\;$$

Where *k*_2_ (minute^−1^) is the PSO rate constant and *q*_t_ and *q*_e_ (mg g^−1^) are the quantity of the dye in the photocatalyst at time t (minute) and equilibrium, respectively.

By plotting t/qt versus time, a linear fit plot, as shown in Figure 8b, is obtained. The kinetic parameters were calculated and are summarized in Table 1. A high k_2_ (2269.49 minutes^−1^) value implies that the photocatalyst is highly reactive, resulting in fast adsorption and degradation, indicating suitability for large‐scale applications. The *q*_e_ (61.31 mg g^−1^) value obtained equally reflects the ability of the synthesized photocatalyst to handle a substantial amount of dye. A high R_2_ value (0.95) supports that the chemisorption is dominant in this degradation.

#### 2.2.10 Intra‐particle Diffusion (IPD) Model

The kinetic data were analyzed using the Weber IPD model to reveal whether IPD was the rate‐limiting stage. The linear fit model of the IPD kinetic model can be given in Equation 3:

(3) $$\;q_{t}=K_{diff}\;\sqrt{t}+C$$

where q_t_ (mg g^−1^) is the amount of adsorbate adsorbed at time t, *k*_diff_ (mg g^−1^.minute^1/2^) is the IPD rate constant, and *C* (mg g^−1^) is the boundary layer effect constant.

The linear fit plot of qt versus $\sqrt{t}$ is represented in Figure 8c, where the R^2^ value of 0.97 was obtained, highlighting the excellent fit to this model. The rate of dye molecules diffusing through the pores of the photocatalyst is represented by *k*_diff_. The intermediate value of *k*_diff_ (2.79 mg g^−1^.minute^1/2^) and the comparatively high value of *C* (0.75 mg g^−1^) show that while diffusion is significant, the system may suffer considerable resistance in the boundary layer, especially at higher dye concentrations. This is characteristic of photocatalytic degradation processes in which the photocatalyst has a large surface area and porosity, allowing diffusion. A greater *C* value would indicate a thicker boundary layer, leading to slower diffusion of dye molecules to the catalyst surface during the initial adsorption step.^[^ ^60^ ^]^

#### 2.2.11 Langmuir‐Hinshelwood (L–H) Kinetic Model

The Langmuir–Hinshelwood (L–H) mechanism is commonly employed to describe solid catalytic reactions. This model is widely recognized for its application in the heterogeneous catalytic degradation of organic pollutants in wastewater.^[^ ^61^ ^]^ The linear form of the L–H model in equation 4 can be used to calculate the relationship between the initial photocatalytic rate and the initial concentration of dye^[^ ^62^ ^]^:

(4) $$\;\frac{1}{r}=\frac{1}{k_{r}.K}\;\frac{1}{C}+\frac{1}{k_{r}}$$

where r is the reaction rate (mg L^−1^.minute), *k*_r_ is the reaction rate constant (L mg^−1^.minute), K is the adsorption equilibrium constant (L mg^−1^), and C is the concentration of reactant (mg L^−1^).

From the linear fit plotting 1/r versus 1/C, the regression coefficient (R^2^) was 0.89, showing sufficient correlation (Figure 8d). K value was 5.46 L mg^−1^, which signifies a moderate affinity of the MB dye molecules for the PC@CL‐10 surface. The reaction rate constant of 0.01 L mg^−1^.minute demonstrates the effectiveness of the catalytic reaction step. These data, as presented in Table 1, suggest that, while the adsorption stage is reasonably effective, the reaction step on the catalytic surface may be the rate‐limiting element, providing insights for further optimization of the photocatalytic process.

The kinetic analysis of MB dye degradation using PC@CL‐10 revealed distinct mechanistic insights through four evaluated models. The IPD approach exhibited the strongest correlation (R^2^ = 0.97), emphasizing diffusion through the mesoporous framework of the material as a critical factor. While PFO (R^2^ = 0.95) and PSO (R^2^ = 0.95) models both showed substantial agreement with experimental data, their parameters indicated different dominant processes‐ physical adsorption for PFO versus surface‐level chemical interactions for PSO, evidenced by a higher rate constant (k₂). However, based on the IPD nonzero intercept parameter (C) further suggested measurable boundary layer effects during mass transfer. In contrast, the L–H model showed a comparatively weaker fit (R^2^ = 0.89), implying limited applicability for describing this particular catalytic system. These collective findings support a hybrid degradation pathway where initial surface adsorption via chemisorption precedes subsequent diffusion‐controlled steps within the porous architecture of PC@CL‐10.

#### 2.2.12 Possible Degradation Mechanism

**Figure 9:**
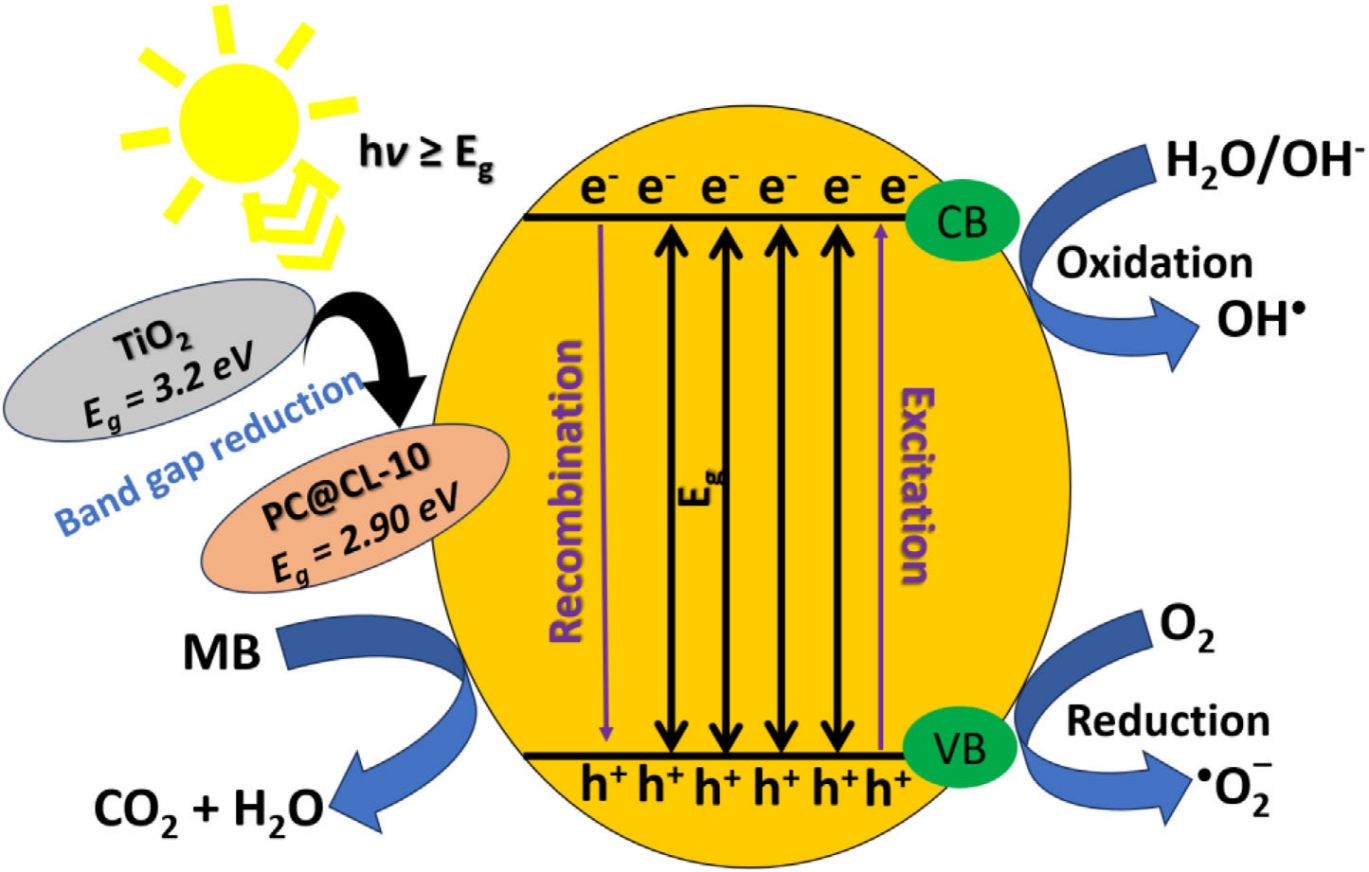
Plausible photocatalytic degradation of MB using PC@CL‐10

The plausible photocatalytic degradation of MB dye under UV illumination with the prepared photocatalyst is depicted in Figure 8. The band gap for PC@CL‐10 was found to be 2.90 eV (refer to supplementary Figure S3 for PL spectra). Compared to TiO_2_ (∼3.2 eV), the reduced band gap significantly improves the photocatalytic degradation of dyes by enhancing visible light absorption and promoting efficient charge separation.^[^ ^63^ ^]^ CL‐10 forms heterojunctions with titania that alter the band structure, resulting in a lower band gap. A band gap of 2.90 eV enables the photocatalyst to absorb light, enhancing its ability to harness a broader portion of the solar spectrum. Thus, PC@CL‐10 exhibits superior efficiency in UV‐driven photocatalysis due to its ability to produce more electron–hole pairs when exposed to UV light. This process promotes e⁻ from the valence band (VB) to the conduction band (CB), resulting in the formation of electron–hole pairs and leaving behind h⁺ in the VB. Energy transfer to CL‐CNPs (CL‐10) occurs as they function as electron acceptors due to their excellent electrical conductivity. Photogenerated electrons from TiO_2_ move to the carbon surface, effectively minimizing electron–hole recombination (Figure 9).

$$\mathrm{Ti}O_{2}+h\nu \to \mathrm{Ti}O_{2}\;(e^{-}+h^{+})$$

$$e^{-}\;(\mathrm{Ti}O_{2})\to e^{-}\;\left(\text{CL-10}\right)$$

$$O_{2}+e^{-}\to \cdot {O_{2}}^{-}$$

$$h^{+}+H_{2}O\to \cdot \mathrm{OH}+H^{+}$$

MB+·OH→Oxidation

$${\mathrm{MB}+\cdot O}_{2}^{-}\to \mathrm{Reduction}$$

$${\mathrm{MB}+\cdot \mathrm{OH}/\cdot O}_{2}^{-}\to \mathrm{Degraded}\;\mathrm{products}\;(CO_{2}+H_{2}O)$$

Formation of ROS as the transferred electrons react with dissolved oxygen in water, producing superoxide radicals (**·**O_2_⁻). The above‐mentioned reactions show these changes. At the same time, holes in the VB of TiO_2_ react with water or hydroxide ions, generating hydroxyl radicals (**·**OH). The **·**OH and **·**O_2_⁻ radicals are highly reactive and target MB molecules, disrupting the chromophore structure and breaking it down into smaller, nontoxic products such as CO_2_, H_2_O, and inorganic ions (refer to supplementary Figure S6 for a plausible degradation sketch). Because of their large surface area and mesoporous nature, CL‐10 suppresses electron–hole recombination and enhances MB adsorption on the photocatalyst surface. This facilitates localized degradation at the PC@CL‐10 (carbon‐TiO_2_) interface, boosting the overall photocatalytic efficiency. The synergistic effect of TiO₂ and carbon enhances electron transfer, reduces recombination, and increases the production of ROS, leading to faster and more effective photocatalytic degradation.

## 3 Experimental Section

### 3.1 Preparation of CNPs (CL‐10)

The synthesis of CNPs was achieved from a biowaste precursor using one‐pot pyrolysis (Scheme 1a). Coffee (*Coffea canephora*) leaves were dried and ground into a coarse powder using a mixer. The resulting powder was sieved through a 75 µm mesh to achieve a uniform particle size. Pyrolysis was performed by heating the precursor to 1000 °C in a quartz tube furnace under a continuous nitrogen gas flow (150 mL/cm^3^/minute) to ensure an inert atmosphere throughout the process. The pyrolyzed material was rinsed with 0.1 N HCl, followed by washing with distilled water, and subsequently, before being used for further studies.

### 3.2 Preparation of photocatalyst (PC@CL‐10)

Commercially available Digussa  TiO_2_ P25 was first activated using a 5 M NaOH solution at room temperature for about 2 hours. The powder was filtered, washed with deionized water, and dried in the oven for 3 hours at 100 °C. In a dried and clean mortar, CL‐CNPs (CL‐10) of 100 mg and TiO_2_ of 20 mg were crushed in one direction for about 30 minutes. The obtained powder was then taken in 10 mL of 95% ethanol and stirred for 30 minutes to obtain a uniform mixture and to achieve surface activation. The mixture was filtered to get a fine powder and was annealed in an oven for 2 hours at 160 °C. This was further characterized and used for photocatalytic degradation. The synthesis is illustrated schematically in the supplementary information, Scheme 1b.

### 3.3 Assessment of photocatalytic activity

The photocatalytic degradation of the dye was carried out in a photocatalytic triple‐jacketed reactor setup. The chamber was equipped with a UV lamp source to simulate the irradiation environment. The experiments were carried out with 100 mL of MB dye solution at a specific dye concentration (2.5–10 ppm). For each trial, 0.05 g/L to 0.20 g/L of the synthesized photocatalyst (PC@CL‐10) was added to the solution, and the suspension was stirred continuously using a magnetic stirrer set at a constant rate to ensure proper mixing and prevent catalyst sedimentation. The experiments were also carried out at different pH levels (acidic, native, basic). The absorbance was noted at every 15‐minute interval to monitor the degradation efficiency and was analyzed through a UV‐Vis spectrophotometer. The absorbance at a maximum wavelength of 660 nm for MB was noted to quantify the decrease in dye concentration. All photocatalytic degradation experiments were conducted under consistent conditions. An estimated error margin was applied to degradation efficiency values based on photocatalyst dispersion, dye concentration control, instrumental precision, and typical experimental variability. The photocatalytic performance was evaluated by the percentage of dye degradation, which was obtained using the following equation 5:

(5) $$Degradation\;\left(\%\right)=\frac{Co-Ct}{Co}\;\times 100\;$$

*C*_o_ and *C*_t_ are the initial and dye concentrations at time (t), respectively.

The specific experimental procedures of characterizations, measurements, and photothermal performance measurements were listed in the supplementary information.

## 4 Conclusion

In this study, CNPs (CL‐10) derived from coffee leaf biowaste were synthesized via pyrolysis at 1000 °C and combined with TiO_2_ to create the photocatalyst (PC@CL‐10). Photocatalytic degradation of MB dye achieved a maximum efficiency of 99% under UV light within 240 minutes. FE‐SEM images confirmed morphological changes that occurred with the addition of TiO_2_ in PC@CL‐10. FTIR, XPS, Raman, and XRD analysis revealed the incorporation of TiO_2_ into CL‐10. Compared to CL‐10 alone, PC@CL‐10 demonstrated significantly enhanced degradation performance due to the synergistic effect of TiO_2_ and CNPs by reducing the band gap to 2.90 eV. The plausible mechanism was elucidated, indicating the effective photocatalytic degradation of dye with the prepared photocatalyst due to electron transfer and the generation of ROS. Kinetic studies suggested that the degradation followed the IPD model, highlighting diffusion as a key mechanism. Additionally, the photocatalyst exhibited excellent recyclability, retaining over 90% efficiency for six cycles, making it a promising material for sustainable wastewater treatment. These characteristics differ from traditional homogeneous methods, highlighting a commitment to environmentally friendly practices. Future research could aim at improving the efficiency of photocatalysts under visible light and testing their performance on real industrial wastewater with complex dye mixtures. Future work will focus on identifying intermediate degradation products and optimizing catalyst regeneration protocols to minimize surface fouling and enhance long‐term photocatalytic stability. Scaling up the process and developing continuous‐flow systems would facilitate the transition from laboratory experiments to industrial applications. Integrating PC@CL‐10 with other photocatalysts or adsorbents may enhance its selectivity and effectiveness. Conducting a life cycle assessment would offer crucial insights into its environmental impact and assess the long‐term sustainability of using this biowaste‐derived photocatalyst for large‐scale water treatment.

## Conflict of Interest

The authors declare no conflict of interest.

## Supporting information



Supporting Information

## Data Availability

The data that support the findings of this study are available from the corresponding author upon reasonable request.
