# Supplementary material for: Efficient Photocatalytic Degradation of Methylene Blue From Aqueous Solution Using Hybrid Biomass‐Derived Nanostructured Carbon‐TiO2 Photocatalyst
Source: Chemistry. 2025 Jul 17;31(44):e202501564. doi: 10.1002/chem.202501564 (PMC12336747; doi:10.1002/chem.202501564)
Supplement: Supplementary file 1 — Supporting Information [file CHEM-31-e202501564-s001.docx]

*Supplementary information*

**Efficient Photocatalytic Degradation of Methylene Blue from Aqueous Solution Using Hybrid Biomass-Derived Nanostructured Carbon-TiO_2_ Photocatalyst**

Aman Sharma ^[a,b]^, Jyothi M. Shivanna ^[c]^, Navneet K. Gupta ^[d]^, Prashanth W. Menezes ^[e,f]^*, Gurumurthy Hegde ^[a,b]^**


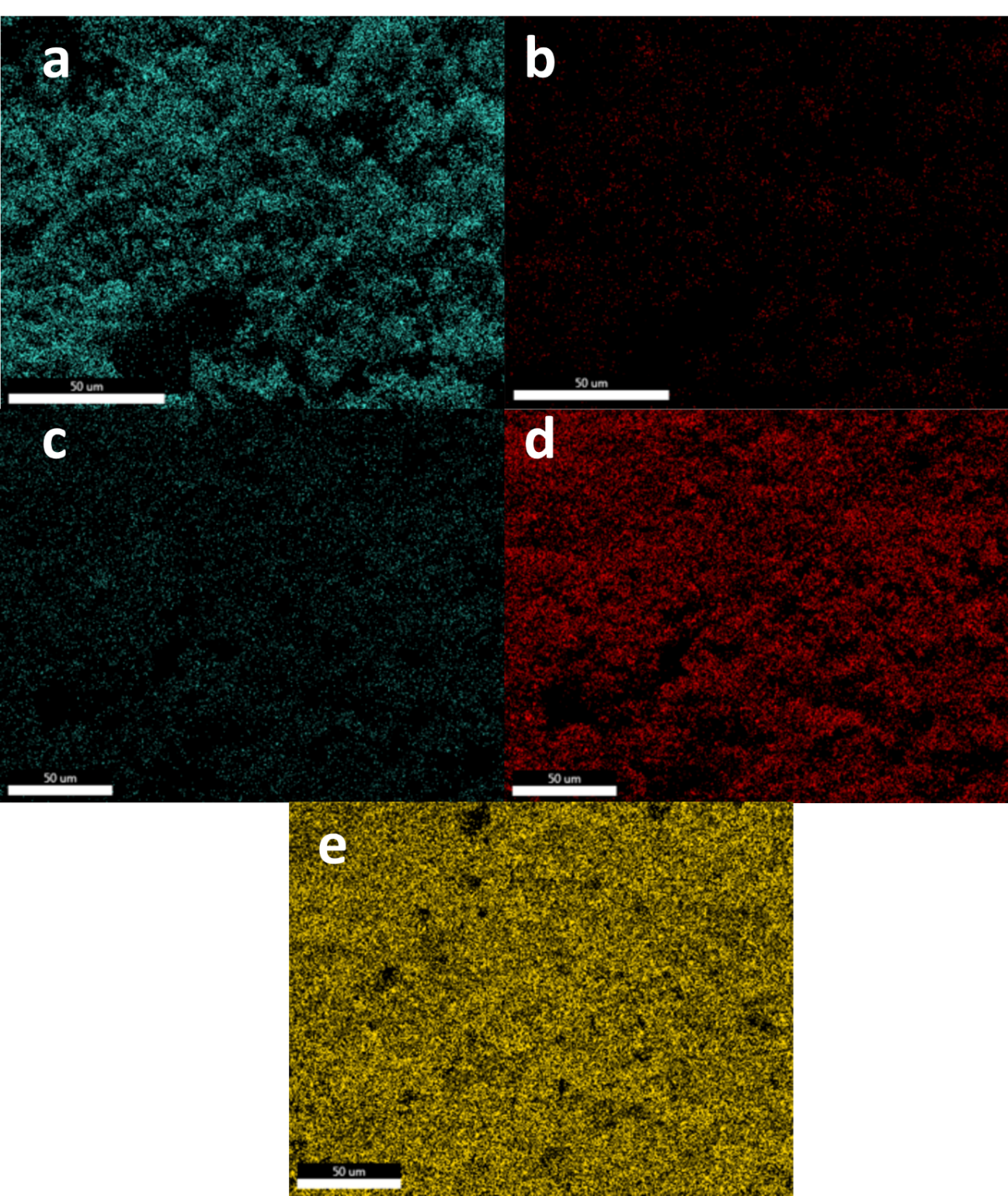


**Figure S1.** EDS mapping of a) Carbon, b) Oxygen in CL-10; c) Carbon, d) Oxygen, and e) Titanium in PC@CL-10.


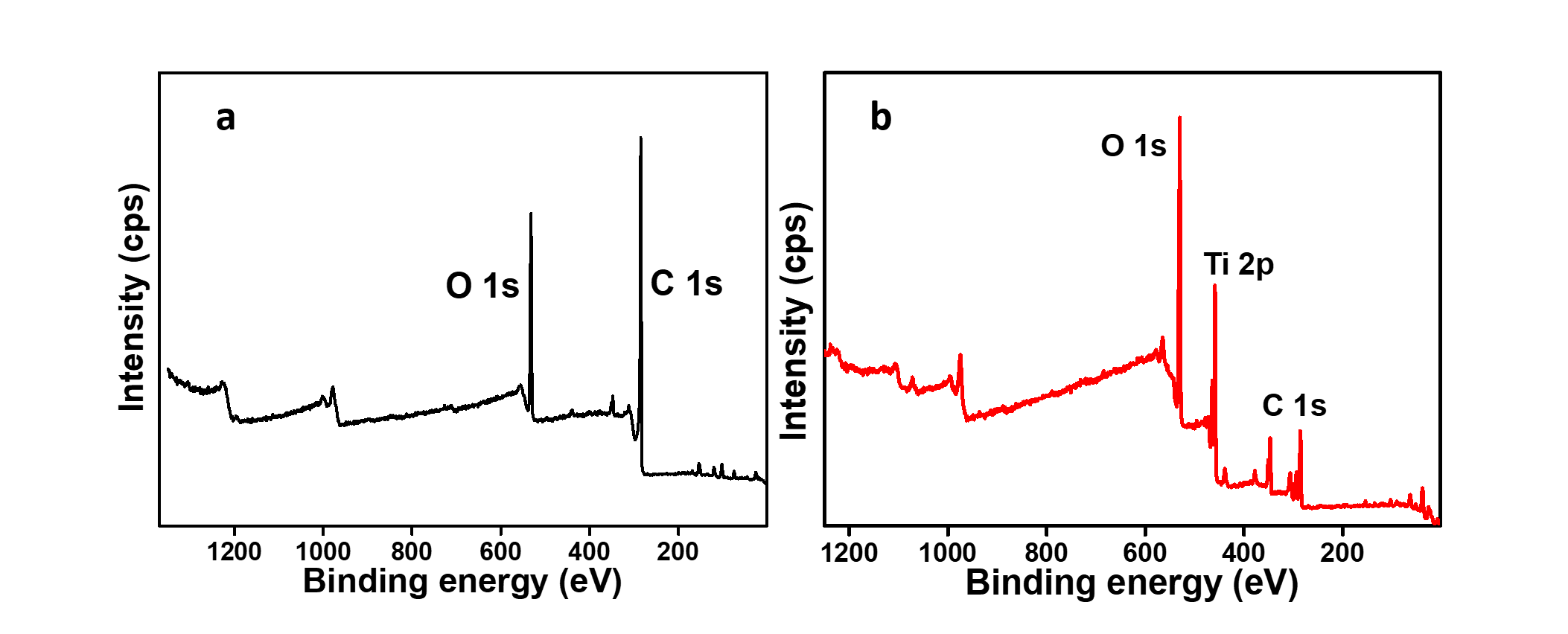


**Figure S2**. XPS survey spectra for a) CL-10 and b) PC@CL-10


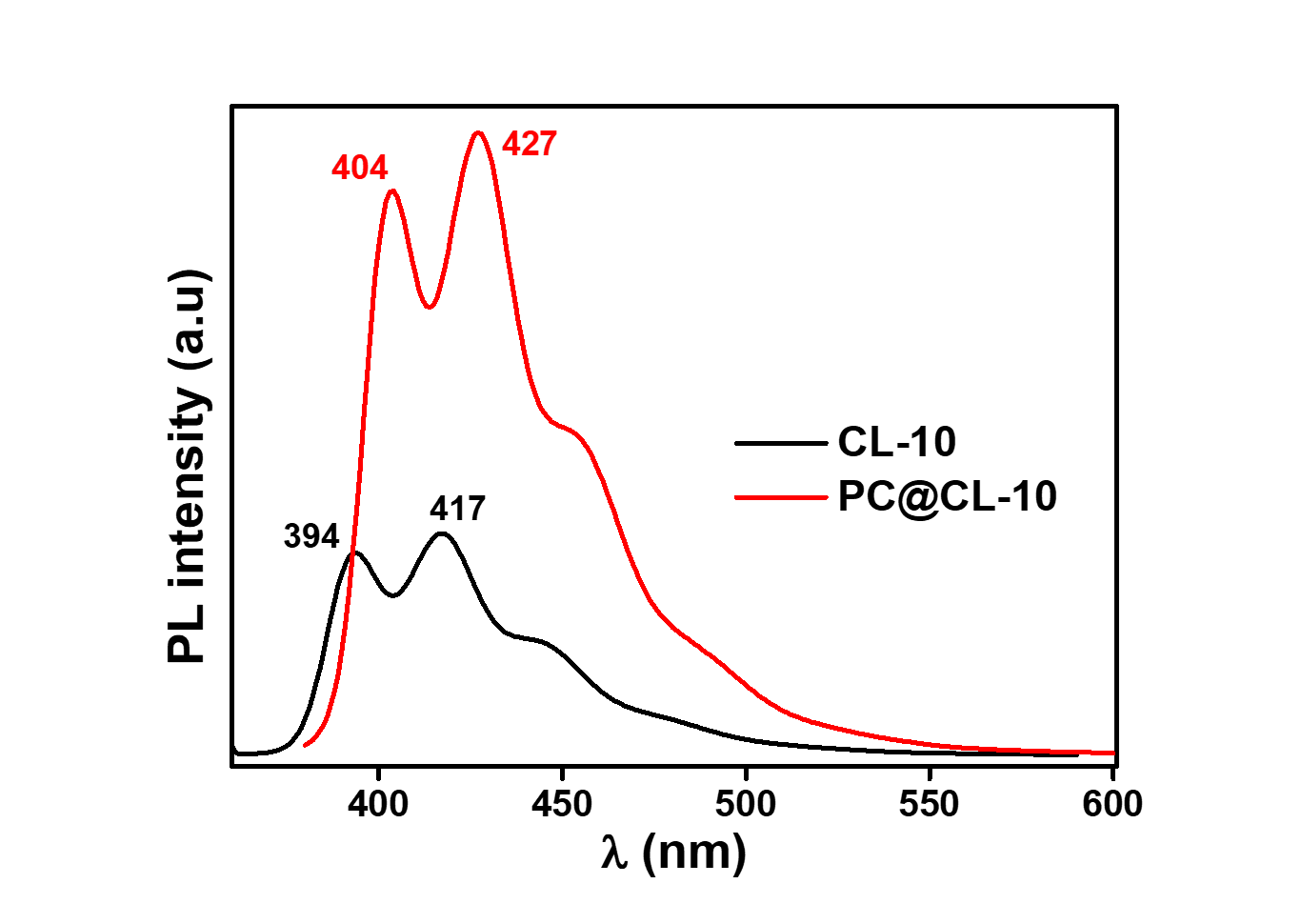


**Figure S3.** PL spectra of CL-10 and PC@CL-10.


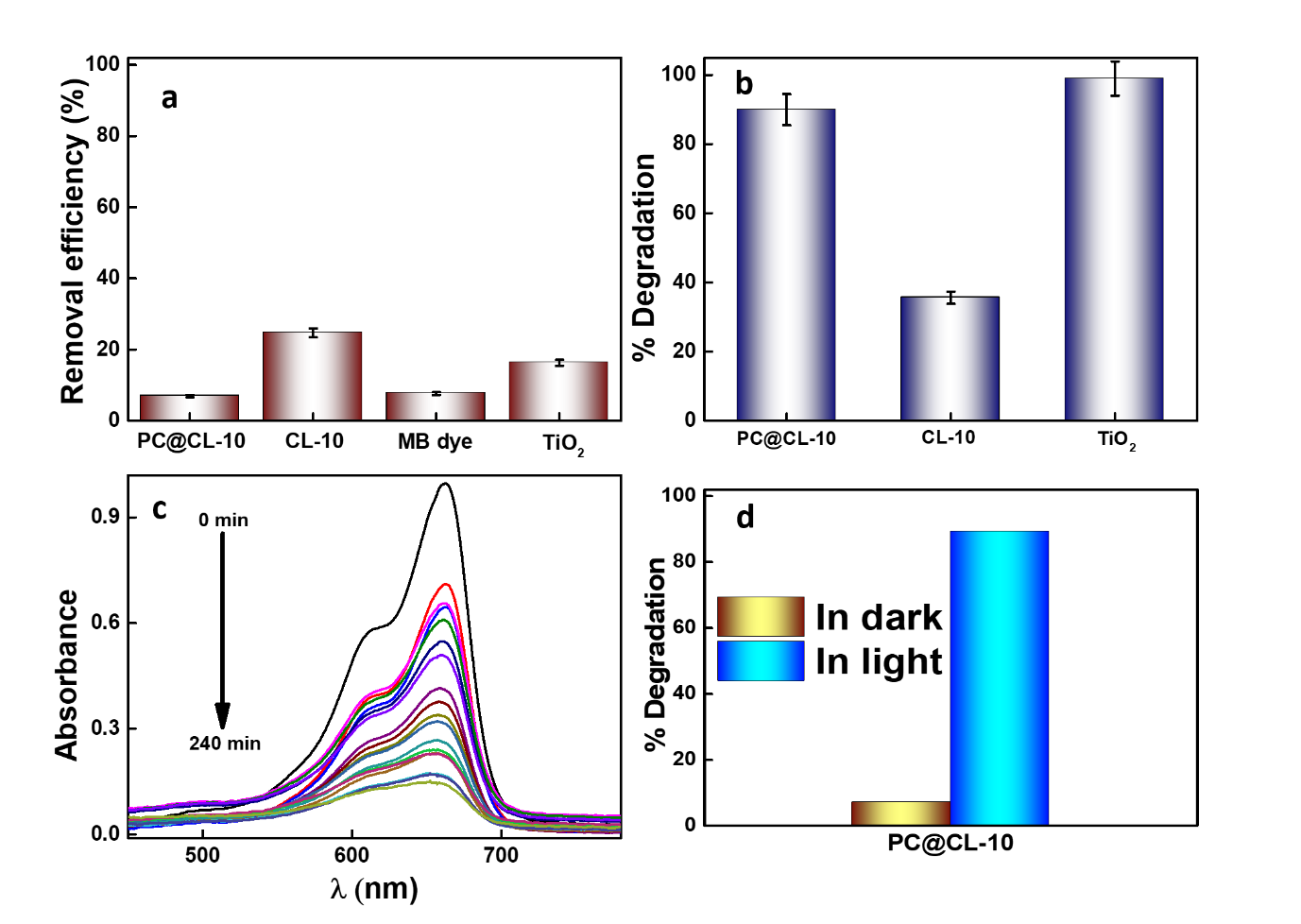


**Figure S4.** a) Removal efficiency of MB dye in dark conditions at 240 minutes; b) Degradation efficiency of MB dye under light illumination at 240 minutes (TiO_2_ in 60 minutes); c) UV absorbance plot for MB dye degradation over 240 minutes of ; d) Bar graph comparison of the prepared photocatalyst(PC@CL-10) under light and dark conditions at 240 minutes


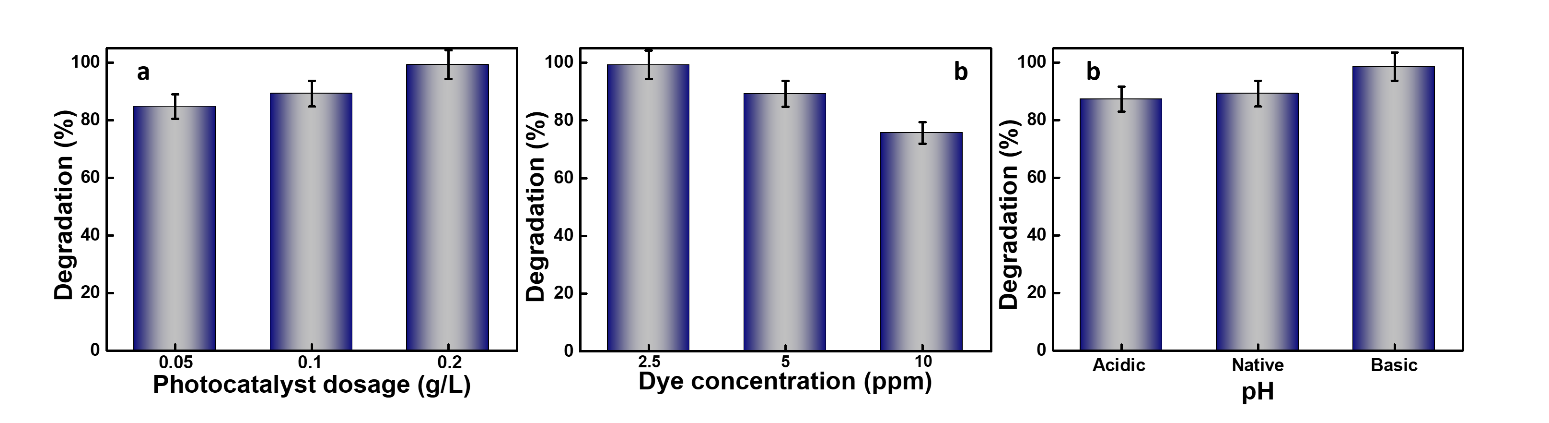


**Figure S5.** Bar graph indicating degradation efficiency at 240 minutes of light irradiation for different a) photocatalyst dosage, b) dye concentration, c) pH


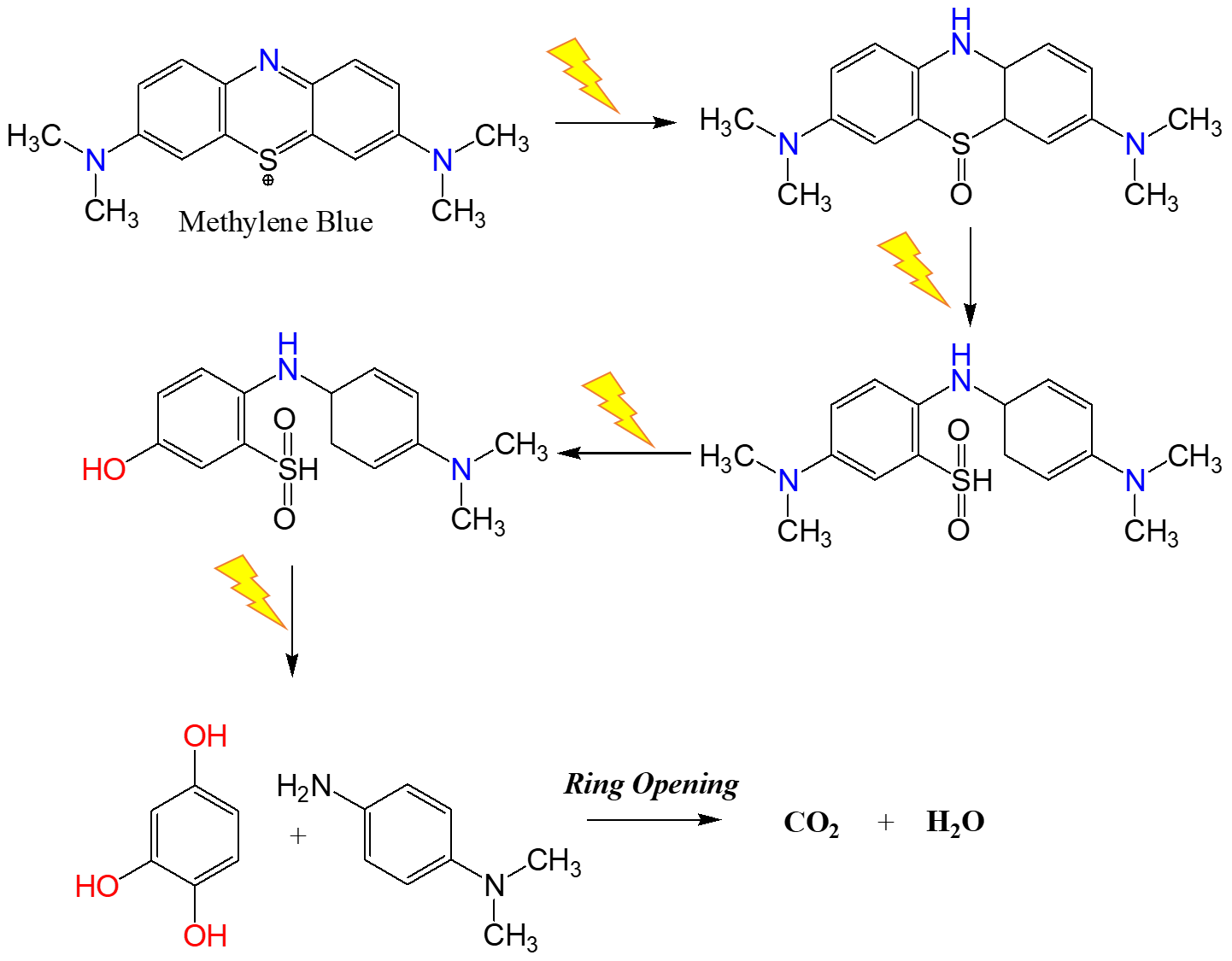


**Figure S6.** Sketch showing the plausible degradation products for the photocatalytic degradation of MB dye with TiO_2_-carbon composite

**Materials**

The biowaste of fallen robusta coffee leaves was sourced from the Coorg, Western Ghats, and was used in this study. MB and Congo Red (CR) dye were obtained from Sigma Aldrich Pvt. Ltd., while sodium hydroxide (NaOH) and hydrochloric acid (HCl) were purchased from CDH and Thomas Baker, respectively. The pyrolysis of the biowaste was conducted in a tube furnace supplied by NoPo Nanotechnologies, India. The pH of the solutions was monitored using a pH meter (Labman Scientific Instruments). A magnetic stirrer thoroughly mixed the photocatalyst and dye at a controlled stirring speed. After treatment, the dye solution with photocatalyst was separated from the solution using a microprocessor-based centrifuge. For regeneration studies, ethanol was used as the solvent. Millipore-distilled water was used throughout the experiments to ensure consistency in all studies.

**Characterizations**

The surface morphology of the as-synthesised nanoparticles and the photocatalyst was analyzed using a scanning electron microscope (SEM) from JOEL JSM-IT300, Japan. The crystallinity of the nanoparticle and the photocatalyst was assessed using X-ray diffraction (XRD) patterns from the MiniFlex 600 Rigaku, Japan, employing Cu-Kα radiation. The chemical structure and functional groups were identified via the Fourier transform infrared (FTIR) approach with IRSpirit-L from Shimadzu, Japan. Brunauer-Emmett-Teller (BET) with BJH pore size distribution was obtained by Quanta Chrome Instruments, AutosorbiQ, and ASiQwin to elucidate surface area and pore characteristics. Raman spectra were examined using a Renishaw Raman microscope to understand the crystalline structure. The absorbance of the MB dye was measured using a UV-visible spectrophotometer. X-ray photoelectron spectroscopy (XPS) was performed to obtain the binding energy of elements using the Axis Ultra Model, Shimadzu, Japan. Spectro Fluorophotometer RF-6000, Shimadzu, Japan, was used to evaluate the photoluminescence property and calculate the bandgap.

**
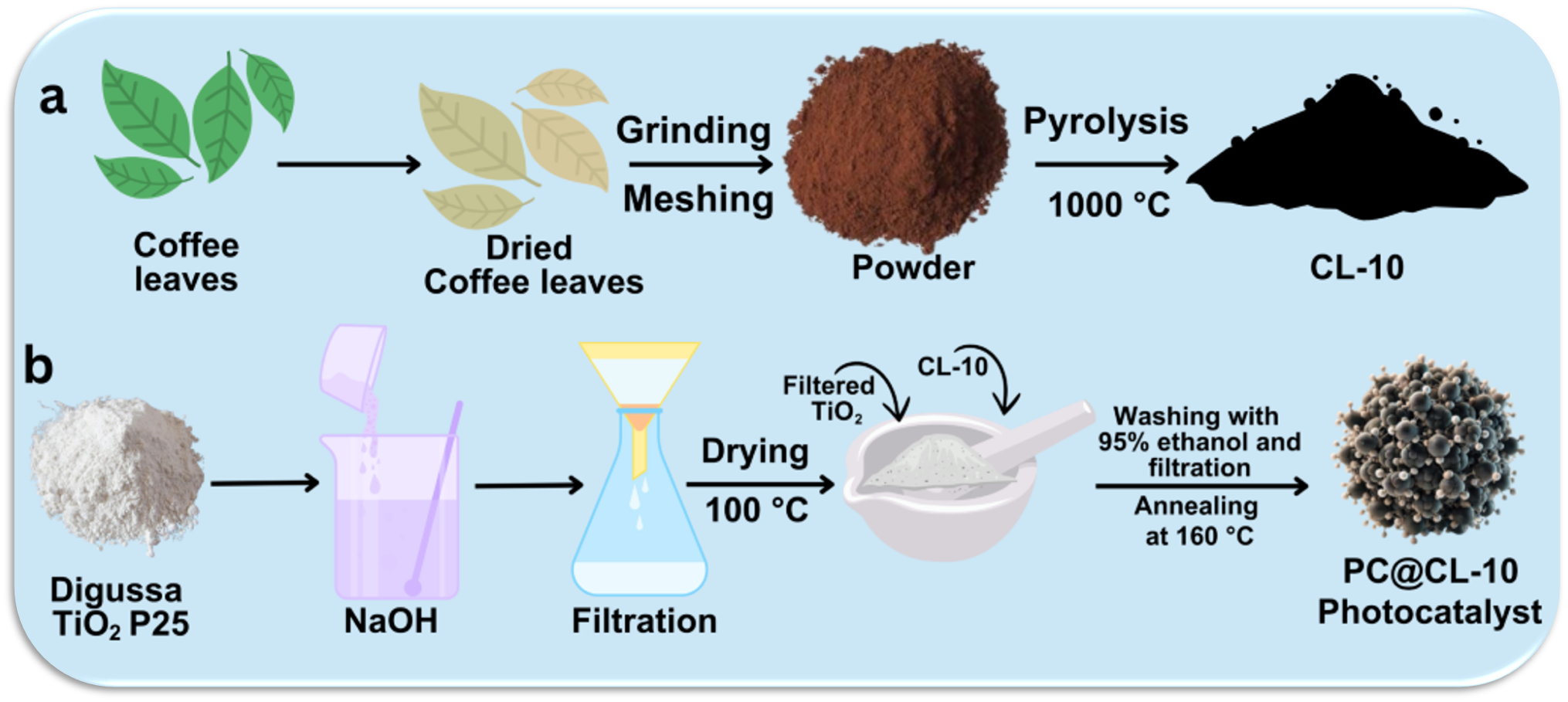
Scheme S1.** Schematic representation of a) synthesis of CL-10 and b) preparation of photocatalyst

**Table S1.** Comparison of Recent TiO₂-Carbon Photocatalysts for Dye Degradation

| **Dye** | **Dye concentration (ppm)** | **Photocatalyst and synthesis route** | **Photocatalyst dosage**  **(g/L)** | **Degradation efficiency (%)** | **Time (min)** | **Ref.** |
| --- | --- | --- | --- | --- | --- | --- |
| RhB | 10 | TiO_2_/SWCNT (wet impregnation) | 0.5 | 100 | 60 | [[1]](https://paperpile.com/c/E17smR/UNpc) |
| MB | 20 | Cotton stalk Carbon-TiO_2_/g-C_3_N_4_ | 1 | 92.6 | 90 | [[2]](https://paperpile.com/c/E17smR/K5h0) |
| MB | 10 | CNs@TiO_2_ (using carboxymethyl starch via chemical modifications) | 2 | 88 | 180 | [[3]](https://paperpile.com/c/E17smR/hkLQ) |
| Reactive Red 120 | 50 | AC@TiO_2_  (Pistachio-shell-derived activated carbon at 800 ℃ and KOH activation) | 5 | 95 | 50 | [[4]](https://paperpile.com/c/E17smR/2s8H) |
| MB | 10 | AC@TiO_2_ (Coconut shell activated carbon with NaOH activation) | 0.33 | 96.6 | 60 | [[5]](https://paperpile.com/c/E17smR/lcxz) |
| **MB** | **2.5** | **PC@CL-10 (coffee leaves derived carbon from pyrolysis at 1000 ℃)** | **0.1** | **99** | **240** | **Present work** |

**References**

[1] [C. Abreu-Jaureguí, L. Andronic, A. Sepúlveda-Escribano, J. Silvestre-Albero, *Environ. Res.* **2024**, *251*, 118672.](http://paperpile.com/b/E17smR/UNpc)

[2] [T. Cui, Y. Zhang, M. Tian, Y. Yan, G. Zhang, T. Zhang, J. Zhao, J. Jiang, *Diam. Relat. Mater.* **2024**, *144*, 111028.](http://paperpile.com/b/E17smR/K5h0)

[3] [Y. A. S. Hameed, A. M. Munshi, M. A. Alsharif, A. M. Alsharari, R. Pashameah, D. M. Alenazy, N. Alkhathami, N. M. El-Metwaly, *Appl. Water Sci.* **2025**, *15*, 77.](http://paperpile.com/b/E17smR/hkLQ)

[4] [Y. G. Alghamdi, B. Krishnakumar, M. A. Malik, S. Alhayyani, *Polymers (Basel)* **2022**, *14*, 880.](http://paperpile.com/b/E17smR/2s8H)

[5] [P. H. Le, T. T. T. Vy, V. Van Thanh, D. H. Hieu, Q.-T. Tran, N.-V. T. Nguyen, N. N. Uyen, N. T. T. Tram, N. C. Toan, L. T. Xuan, L. T. C. Tuyen, N. T. Kien, Y.-M. Hu, S.-R. Jian, *Micromachines (Basel)* **2024**, *15*, 714.](http://paperpile.com/b/E17smR/lcxz)
